# Heterogeneous beta-catenin activation is sufficient to cause hepatocellular carcinoma in zebrafish

**DOI:** 10.1242/bio.047829

**Published:** 2019-10-01

**Authors:** Sharanya M. Kalasekar, Srishti Kotiyal, Christopher Conley, Cindy Phan, Annika Young, Kimberley J. Evason

**Affiliations:** 1 Department of Oncological Sciences, Huntsman Cancer Institute, University of Utah, Salt Lake City, UT 84112, USA; 2 Department of Pathology, University of Utah, Salt Lake City, UT 84112, USA

**Keywords:** Zebrafish, Hepatocellular carcinoma, CreLox, Single-cell RNAseq, Beta-catenin

## Abstract

Up to 41% of hepatocellular carcinomas (HCCs) result from activating mutations in the *CTNNB1* gene encoding β-catenin. HCC-associated *CTNNB1* mutations stabilize the β-catenin protein, leading to nuclear and/or cytoplasmic localization of β-catenin and downstream activation of Wnt target genes. In patient HCC samples, β-catenin nuclear and cytoplasmic localization are typically patchy, even among HCC with highly active *CTNNB1* mutations. The functional and clinical relevance of this heterogeneity in β-catenin activation are not well understood. To define mechanisms of β-catenin-driven HCC initiation, we generated a Cre-lox system that enabled switching on activated β-catenin in (1) a small number of hepatocytes in early development; or (2) the majority of hepatocytes in later development or adulthood. We discovered that switching on activated β-catenin in a subset of larval hepatocytes was sufficient to drive HCC initiation. To determine the role of Wnt/β-catenin signaling heterogeneity later in hepatocarcinogenesis, we performed RNA-seq analysis of zebrafish β-catenin-driven HCC. At the single-cell level, 2.9% to 15.2% of hepatocytes from zebrafish β-catenin-driven HCC expressed two or more of the Wnt target genes *axin2*, *mtor*, *glula*, *myca* and *wif1*, indicating focal activation of Wnt signaling in established tumors. Thus, heterogeneous β-catenin activation drives HCC initiation and persists throughout hepatocarcinogenesis.

## Abstract (precis)

**Summary:** Here we show that expression of activated β-catenin in a subset of early hepatocytes is sufficient to drive liver tumorigenesis. Heterogeneity in β-catenin activation continues during HCC progression.

## INTRODUCTION

Liver cancer is the seventh most commonly occurring cancer in the world and the third highest contributor to cancer-related mortality (Ghouri et al., 2017). Hepatocellular carcinoma (HCC) comprises over 90% of all primary liver cancers (Lin et al., 2017). Approved chemotherapeutic strategies against HCC include multi-kinase inhibitors and immune-checkpoint inhibitors. However, patient response to these drugs and improvement in survival are marginal (Zhu and Hoshida, 2018). Genome-wide analyses of clinical HCC samples reveal a range of functional molecular aberrations that can be grouped into distinct molecular subclasses (Chiang et al., 2008). A substantial subset of HCC (13–41%) is defined by activating mutations in the gene encoding β-catenin (*CTNNB1*) (Khalaf et al., 2018).

β-catenin is a multifunctional protein with two cellular pools: one pool acts in the canonical Wnt signaling pathway, while the other pool functions independently of Wnt, connecting cadherins to the actin cytoskeleton as part of adherens junctions (Monga, 2015). Activation of the Wnt pathway triggers cytoplasmic accumulation of β-catenin, followed by nuclear localization and activation of downstream target genes that function in a variety of processes including embryonic development and hepatoblast differentiation, proliferation and survival (Giles et al., 2003; Goessling et al., 2008; Laurent-Puig et al., 2001; Russell and Monga, 2018; Sanyal et al., 2010; So et al., 2013; Tan et al., 2008). Aberrant activation of the Wnt pathway is associated with several types of cancer including endometrial cancer, melanoma, colorectal cancer and HCC (Kim and Jeong, 2019). Oncogenic mutations stabilize the β-catenin protein, leading to nuclear and/or cytoplasmic localization of β-catenin and activation of Wnt target genes (Austinat et al., 2008; de La Coste et al., 1998). Given that β-catenin mutations act as drivers or initiating events in HCC (Laurent-Puig et al., 2001; Nault et al., 2013; Torrecilla et al., 2017; Zucman-Rossi et al., 2006), and that β-catenin accumulates in the nucleus or cytoplasm in half of all cancer cases (Shang et al., 2017), it is imperative to define the pathogenesis of β-catenin-driven cancer using vertebrate models.

Researchers are beginning to understand the importance of intratumor heterogeneity in the pathogenesis and treatment of cancer, including HCC (Zhu and Hoshida, 2018). Eighty-seven percent of HCC show intratumor heterogeneity with respect to morphology, immunohistochemistry and/or mutational status of *CTNNB1* or *TP53* (Friemel et al., 2015). In β-catenin-driven HCC, *CTNNB1* mutations are generally ubiquitous within each tumor (Torrecilla et al., 2017), but nuclear and cytoplasmic localization of β-catenin is heterogeneous in both animal models (Evason et al., 2015; Qiao et al., 2018) and patients (Friemel et al., 2015; Rebouissou et al., 2016). For example, we previously reported that hepatocyte-specific expression of activated β-catenin in zebrafish [*Tg(fabp10a*:*pt-β-cat)*] leads to HCC in 78% of animals by 6 months post fertilization (mpf) (Evason et al., 2015). These zebrafish HCC share morphologic and genetic similarities with human HCC (Evason et al., 2015). Although *Tg(fabp10a*:*pt-β-cat)* zebrafish contain the transgene encoding activated β-catenin in all hepatocytes, β-catenin nuclear and/or cytoplasmic staining is only observed in scattered cells in these zebrafish HCC (Evason et al., 2015). This heterogeneity is also seen in patients; even among HCC with similar, highly active *CTNNB1* mutations affecting the D32-S37 region of exon 3, β-catenin still displays heterogeneous expression/localization patterns ranging from absent to rare to frequent nuclear staining (Rebouissou et al., 2016). The clinical and functional significance of heterogeneous β-catenin localization in HCC progression are unclear.

One possible explanation for this variable β-catenin localization is that only very low levels of activated β-catenin, undetectable by immunohistochemistry, are required to drive target gene expression. Supporting this hypothesis is that diverse β-catenin mutations and β-catenin nuclear/cytoplasmic localization correlate with expression of the β-catenin target gene glutamine synthetase (GS) and strong, diffuse immunohistochemical staining for GS (Adebayo Michael et al., 2019; Audard et al., 2007; Austinat et al., 2008; Cieply et al., 2009; Friemel et al., 2015; Rebouissou et al., 2016; Zucman-Rossi et al., 2006). However, Hale et al. found that 83% (39/47) of HCC with diffuse GS staining lacked exon 3 β-catenin mutation (Hale et al., 2016), and Austinat et al. reported that 71% (25/35) of immunohistochemically GS-positive tumors had no detectable β-catenin mutation (Austinat et al., 2008). These results imply that GS may be turned on by β-catenin-independent mechanisms. In this case, diffuse GS expression would not necessarily indicate diffuse β-catenin activation. An alternative explanation for the heterogeneous staining is that β-catenin activity is only required in a subset of cells to drive HCC progression through mechanisms that are at least partly non-cell-autonomous.

A related question to the significance of heterogeneous β-catenin in HCC progression is the role of β-catenin in HCC initiation: is activated β-catenin required in most or all hepatocytes to initiate HCC, or is its presence in a subset of hepatocytes sufficient for tumor initiation? In most transgenic animal models of HCC (He et al., 2015; Wrighton et al., 2019), including those driven by activated β-catenin (Evason et al., 2015), the oncogene of interest is present in most or all hepatocytes, but it is not clear if this diffuse expression is a requirement for tumor initiation. In mice, activated β-catenin expression in a progenitor population representing 4% of developing liver cells was sufficient to initiate HCC, although 42% of HCC-bearing mice also had hepatoblastomas (Mokkapati et al., 2014). Using a distinct vertebrate HCC model may help to more definitively determine if activated β-catenin expression in a subset of hepatocytes can initiate HCC.

In this study, we tested two related hypotheses: (1) activated β-catenin in a subset of early hepatocytes is sufficient to initiate HCC; and (2) β-catenin activity is heterogeneous during HCC progression. We used zebrafish to develop a vertebrate model of HCC that allows spatial and temporal control of β-catenin activation and performed single-cell and bulk RNA-sequencing of β-catenin-driven HCC. Our results support the hypothesis that activated β-catenin expression in a small subset of hepatocytes is sufficient to drive HCC initiation and indicate the presence of diverse populations of hepatocytes with heterogeneous Wnt target gene expression during HCC progression.

## RESULTS

### Designing a system of modular switches to turn on β-catenin

**Fig. 1.**
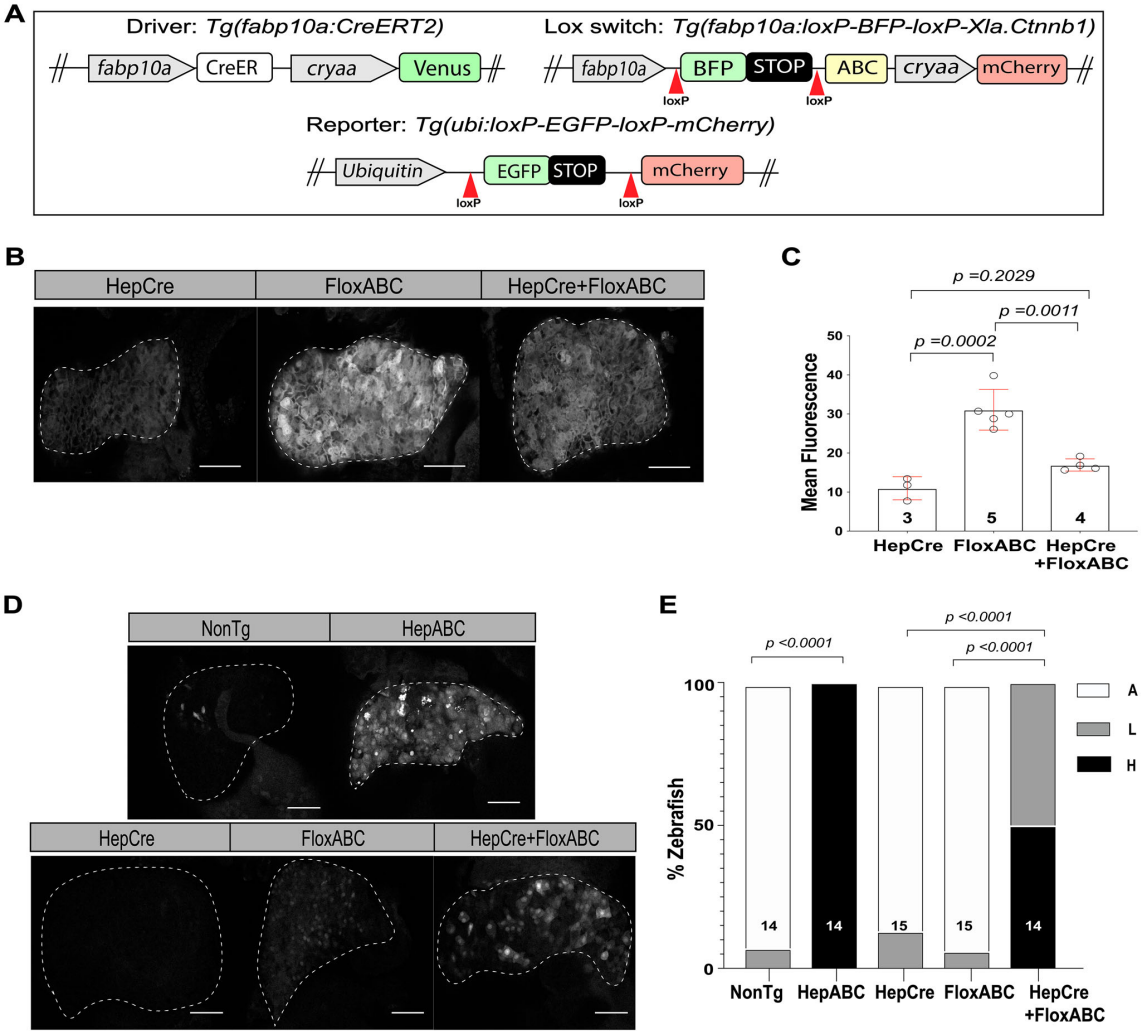
**CreLox system enables switching on activated β-catenin.** (A) Schematics of DNA constructs used in this study. Grey boxes indicate promoters; red arrowheads indicate loxP sites. ABC, Activated β-catenin. (B,C) Switching in *Tg(fabp10a:Cre)* (HepCre)*,Tg(fabp10a:flox-pt-β-cat)* (FloxABC) and *Tg(fabp10a:Cre); Tg(fabp10a:flox-pt-β-cat)* (HepCre+FloxABC) larvae imaged at 5 dpf for BFP expression, detected by immunofluorescence using an anti-GFP antibody. Successful switching is indicated by loss of BFP expression. (B) Representative images. Livers are outlined. Scale bars: 50 µm. (C) Scatter plot with bar graph quantifying switching in terms of mean intensity of GFP fluorescence, ±standard deviation (s.d.). *P*-values derived using Sidak's multiple comparisons test following ordinary one-way ANOVA. *N* values are shown above x axis. Experiment was performed three times, with similar results each time, and representative results are shown. (D,E) Wnt reporter activity in *Tg(fabp10a:Cre)* (HepCre)*,Tg(fabp10a:flox-pt-β-cat)* (FloxABC) and *Tg(fabp10a:Cre); Tg(fabp10a:flox-pt-β-cat)* (HepCre+FloxABC) siblings imaged at 5 dpf alongside *Tg(fabp10a:pt-β-cat)* (HepABC) and non-transgenic siblings (NonTg). All zebrafish contain *7xTCF-Xla.Siam:*mCherry transgene for visualization of Wnt reporter activity by immunofluorescence. (D) Representative images of Wnt reporter activity (*7xTCF-Xla.Siam:*mCherry). Livers are outlined. Scale bars: 50 µm. (E) Stacked bar graphs showing percent of zebrafish with absent (A), low (L, involving <10% of hepatocytes) or high (H, involving >10% of hepatocytes) Wnt reporter activity. *P*-values derived using Fisher's exact test. *N* values are shown above x axis. Three experiments were pooled.

To define mechanisms of β-catenin-driven hepatocarcinogenesis, we sought to turn on activated β-catenin with temporal and spatial control by developing a system of modular switches. We chose a Cre-lox system because this switch produces an irreversible, all-or-none event that occurs on a cell-by-cell basis depending on whether or not Cre-mediated recombination has taken place within that cell or its predecessors. In our system of modular switches, the *Tg(fabp10a:CreERT2)* line is used as a driver (Fig. 1A and Table S1). This transgenic line consists of *CreER^T2^* cDNA, encoding Cre recombinase fused to the modified ligand-binding domain of the human estrogen receptor (ER^T2^) (Branda and Dymecki, 2004; Feil et al., 1997; Metzger and Chambon, 2001), downstream of the hepatocyte-specific *fabp10a* promoter (Her et al., 2003b). The lox-switch part of our modular system consists of the *Tg(fabp10a:loxP-BFP-loxP-Xla.Ctnnb1)* line, hereafter referred to as *Tg(fabp10a:flox-pt-β-cat)*, wherein the *fabp10a* promoter drives expression of activated β-catenin preceded by a blue fluorescent protein (BFP)-STOP cassette flanked by loxP sites (Fig. 1A). In the absence of Cre, transgenic β-catenin remains inactive and BFP is expressed under the control of the *fabp10a* promoter. When Cre is active within a cell, the BFP coding sequence is irreversibly removed, placing activated β-catenin immediately downstream of the *fabp10a* promoter. The resulting *Tg(fabp10a:CreERT2); Tg(fabp10a:flox-pt-β-cat)* system is expected to have spatial control, as activated β-catenin is switched on only in hepatocytes due to the use of the *fabp10a* promoter to drive both components of the switch. We also predicted the system to be temporally controlled by tamoxifen treatment.

We first tested the efficiency of the lox-switch [*Tg(fabp10a:flox-pt-β-cat)*], examining the effect of Cre recombinase on BFP expression and Wnt reporter activity using the previously characterized *Tg(fabp10a:Cre)* zebrafish (Ni et al., 2012) and the Wnt reporter line *Tg(7xTCF-Xla.Siam: mCherry)*, wherein cells with active Wnt signaling show nuclear mCherry expression (Moro et al., 2012). We found that BFP expression was significantly reduced and Wnt reporter activity was significantly increased in *Tg(fabp10a:Cre); Tg(fabp10a:flox-pt-β-cat)* zebrafish livers compared to control siblings without Cre recombinase (Fig. 1B–E). These data indicate that *Tg(fabp10a:flox-pt-β-cat)* zebrafish express functional activated β-catenin in their livers in the presence of Cre recombinase.

**Fig. 2.**
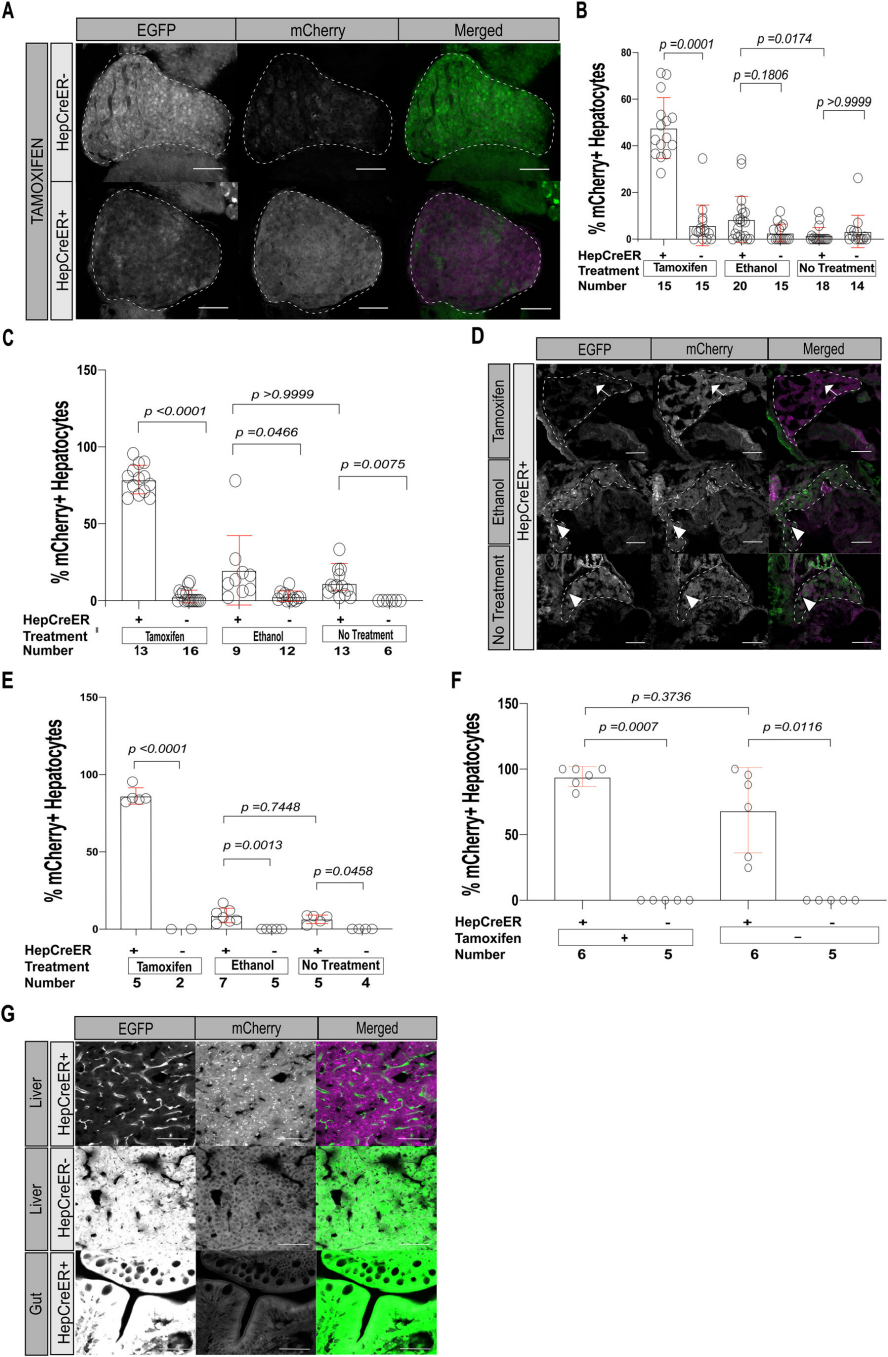
**Analysis of *Tg(ubi:switch)* larvae shows *fabp10a:*CreERT2 leads to significant switching by 10 dpf even in the absence of tamoxifen treatment.** (A,B) Switching in *Tg(ubi:switch)* zebrafish without (HepCreER−) and with (HepCreER+) the *fabp10a:*CreERT2 transgene, incubated with 4-hydroxytamoxifen (tamoxifen), ethanol, or egg water alone (no treatment) from 3 to 6 dpf and imaged at 6 dpf for EGFP and mCherry expression. Successful switching is indicated by loss of EGFP and gain of mCherry expression. (A) Representative images. Scale bars: 50 µm. Livers are outlined. (B) Scatter plot with bar graph quantifying switching in terms of ratio of hepatocytes that switched (mCherry+) relative to the total number of hepatocytes, ±s.d. *P*-values derived using Kruskal–Wallis non-parametric ANOVA followed by Dunn's multiple comparisons test. Graph shows combined data from two experiments. (C,D) Quantification of switching at 10 dpf in *Tg(fabp10a:CreERT2);Tg(ubi:switch)* (HepCreER+) and *Tg(ubi:switch)* (HepCreER−) larvae, incubated with tamoxifen, ethanol, or no treatment from 3 to 6 dpf. (C) Scatter plot with bar graph showing percentage of hepatocytes that switched (mCherry+) relative to the total number of hepatocytes, ±s.d. *P*-values obtained using Kruskal–Wallis non-parametric ANOVA followed by Dunn's multiple comparisons test. Graph represents combined data values from three experiments. (D) Representative images of HepCreER+ larvae. In TAM-treated zebrafish (top panels), most hepatocytes show switching (loss of EGFP and gain of mCherry expression); arrows indicate hepatocytes without switching. HepCreER+ zebrafish not treated with TAM (middle and bottom panels) show occasional cells with switching (arrowheads). Livers are outlined. Scale bars: 50 µm. (E) Quantification of switching at 20 dpf in HepCreER+ and HepCreER− larvae, incubated with tamoxifen, ethanol, or no treatment from 3 to 6 dpf. Scatter plot with bar graph shows percentage of hepatocytes that switched (mCherry+) relative to the total number of hepatocytes, ±s.d. *P*-values obtained using Sidak's multiple comparisons test following ordinary one-way ANOVA. This experiment was performed once. (F,G) Quantification of switching in adult (3 mpf) HepCreER+ and HepCreER− zebrafish, incubated with tamoxifen, ethanol, or no treatment from 3 to 6 dpf. (F) Scatter plot with bar graph shows the percentage of hepatocytes that switched (mCherry+) relative to the total number of hepatocytes, ±s.d. Graph represents combined data values from two experiments. *P*-values obtained using non-parametric Kruskal–Wallis test followed by Dunn's multiple comparisons test. (G) Representative images of liver (top and middle panels) or gut (bottom panel) cryosections showing complete switching (top panel) or no switching (middle and bottom panels). Scale bars: 40 µm.

To confirm the efficiency of *Tg(fabp10a:CreERT2)* in driving tamoxifen-responsive, irreversible recombination, we employed the previously characterized Cre-dependent reporter line *Tg(ubi:loxP-EGFP-loxP-mCherry)*, hereafter referred to as *Tg(ubi:switch)*, wherein switching can be quantified based on gain of mCherry expression and concurrent loss of EGFP expression (Mosimann et al., 2011). At 6 dpf, we observed mCherry expression in 48% of 4-hydroxytamoxifen (TAM)-treated *Tg(fabp10a:CreERT2);Tg(ubi:switch)* hepatocytes (100% of zebrafish showed mCherry expression in some hepatocytes), significantly more than in TAM-treated *Tg(ubi:switch)* hepatocytes lacking Cre expression (6% of hepatocytes, *P*=0.0001; 73% of zebrafish) (Fig. 2A,B). We did not observe a significant increase in mCherry-expressing hepatocytes at 6 dpf in *Tg(fabp10a:CreERT2);Tg(ubi:switch)* siblings treated with vehicle (ethanol) or incubated in egg water alone (Fig. 2B and Fig. S1). There was no detectable mCherry expression in non-hepatocyte cell types including endothelial cells, biliary cells, or intestinal tissues of *Tg(fabp10a:CreERT2);Tg(ubi:switch)* zebrafish (Fig. 2A and Fig. S1). Thus, *fabp10a:*CreERT2 effectively mediates recombination specifically in hepatocytes in response to TAM treatment.

To determine how tightly TAM controls Cre activity in *Tg(fabp10a:CreERT2)* zebrafish, we quantified the numbers of hepatocytes undergoing switching in *Tg(fabp10a:CreERT2)* larvae at later time points. At 10 dpf, we observed mCherry expression in 79% of TAM-treated *Tg(fabp10a:CreERT2);Tg(ubi:switch)* hepatocytes (100% of zebrafish), significantly more than in TAM-treated *Tg(ubi:switch)* hepatocytes (2.7% of hepatocytes, *P*<0.0001; 37.5% of zebrafish) (Fig. 2C,D, and Fig. S2). Ethanol-treated *Tg(fabp10a:CreERT2);Tg(ubi:switch)* zebrafish also exhibited significantly more switching (20% of hepatocytes; 100% of zebrafish) than the *Tg(ubi:switch)* group (2.8% of hepatocytes, *P*=0.0349; 75% of zebrafish) (Fig. 2C,D, and Fig. S2). In the untreated groups we observed significantly more mCherry expression in *Tg(fabp10a:CreERT2);Tg(ubi:switch)* zebrafish (11.5% of hepatocytes; 100% of zebrafish) than in *Tg(ubi:switch)* siblings (0% of hepatocytes, *P*=0.0056; 0% of zebrafish) (Fig. 2C,D, and Fig. S2). At 20 dpf, the differences were essentially maintained. TAM-treated *Tg(fabp10a:CreERT2);Tg(ubi:switch)* zebrafish again showed significantly higher switching (86% of hepatocytes; 100% of zebrafish) than *Tg(ubi:switch)* siblings (0% of hepatocytes, *P*<0.0001; 0% of zebrafish) (Fig. 2E and Fig. S3). In the ethanol-treated and untreated groups, *Tg(fabp10a:CreERT2);Tg(ubi:switch)* zebrafish showed significantly more switching (8.8% and 6.5% of hepatocytes; 100% and 100% of zebrafish) than *Tg(ubi:switch)* siblings (0% and 0% of hepatocytes, *P*=0.0010 and 0.0346; 0% and 0% of zebrafish) (Fig. 2E and Fig. S3). Variation in percent of switching at 6 dpf, 10 dpf, and 20 dpf may be related to technical differences in quantification at the different time points. This analysis of TAM-independent switching in larval zebrafish reveals that *fabp10a:*CreERT2 leads to a background level of switching in about 9% of larval hepatocytes (mean of untreated and ethanol-treated groups at 6, 10 and 20 dpf), even in the absence of TAM treatment.

To characterize TAM-dependent and TAM-independent switching in adult male and female zebrafish, *Tg(fabp10a:CreERT2);Tg(ubi:switch)* zebrafish and *Tg(ubi:switch)* siblings were raised to 3 mpf and treated with TAM, vehicle, or water only (untreated). One week post-treatment, we observed mCherry expression in 94% of TAM-treated *Tg(fabp10a:CreERT2);Tg(ubi:switch)* hepatocytes (100% of zebrafish), significantly more than in TAM-treated *Tg(ubi:switch)* siblings (0% of hepatocytes, *P*=0.0007; 0% of zebrafish) (Fig. 2F,G). We found that 69% of vehicle-treated and untreated *Tg(fabp10a:CreERT2);Tg(ubi:switch)* hepatocytes (100% of zebrafish), also expressed hepatic mCherry in the liver [*P*=0.0116 compared to *Tg(ubi:switch)* siblings] (Fig. 2F). The percent of hepatocytes that had switched was not significantly different in TAM-treated *Tg(fabp10a:CreERT2);Tg(ubi:switch)* zebrafish than in vehicle-treated and untreated control siblings (*P*=0.3736). In all zebrafish, non-hepatocyte tissues including intestine showed only EGFP expression (Fig. 2G), confirming the hepatocyte specificity of *fabp10a*:CreERT2. Thus, although the majority of hepatocytes have already switched by 3 mpf even in the absence of TAM treatment, TAM treatment of adult *Tg(fabp10a:CreERT2)* zebrafish increases the percentage of switched hepatocytes by 25% (96% minus 69%), enabling switching on β-catenin in a subset of adult hepatocytes.

### Oncogene switching in larval livers leads to HCC in adults

We developed a CreLox system (*Tg(fabp10a:CreERT2);Tg(fabp10a:flox-pt-β-cat)* zebrafish, hereafter referred to as CreLox) that enables switching on activated β-catenin in (1) the majority of larval hepatocytes in response to TAM or (2) a subset (about 9%) of larval hepatocytes in the absence of TAM. We used this CreLox model to test two related hypotheses: (1) switching on activated β-catenin in the majority of larval hepatocytes is sufficient to drive liver tumorigenesis; and (2) switching on activated β-catenin in a subset of larval hepatocytes is sufficient to drive liver tumorigenesis. CreLox zebrafish*,* along with sibling controls lacking either the Cre driver or the lox-switch transgene, were treated with 10 μM TAM or vehicle from 3 dpf to 6 dpf and then maintained under standard conditions until 6 mpf. In parallel under the same husbandry conditions, we maintained *Tg(fabp10a:pt-β-cat)* zebrafish, which carry activated β-catenin under direct control of the *fabp10a* promoter, and non-transgenic sibling controls.

**Fig. 3.**
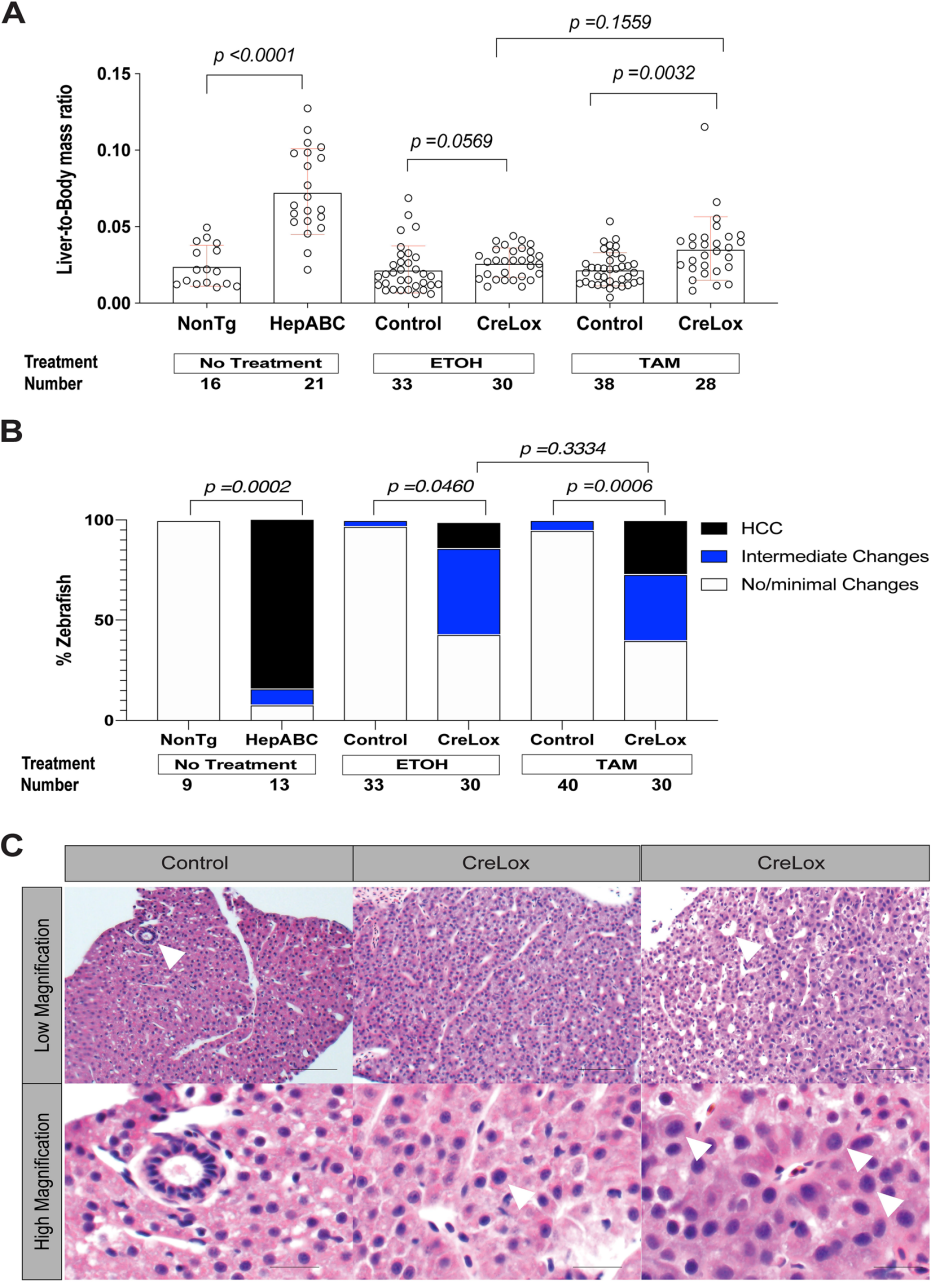
**Switching on activated β-catenin (ABC) in larval zebrafish results in hepatocellular carcinoma (HCC) in adult zebrafish.** CreLox [*Tg(fabp10a:CreER^T2^); Tg(fabp10a:flox-pt-β-cat)*] zebrafish and control siblings lacking either the Cre driver or lox-switch transgene [*Tg(fabp10a:flox-pt-β-cat)* and *Tg(fabp10a:CreER^T2^)*, control], were treated with 4-hydroxytamoxifen (TAM) or vehicle control (ethanol, EtOH) from 3 to 6 dpf. Livers were weighed and examined microscopically 6 months later alongside livers from non-transgenic (NonTg) and *Tg(fabp10a:pt-β-cat)* (HepABC) zebrafish. Data were pooled from two experiments for NonTg and HepABC groups and from five experiments for control and CreLox groups treated with ETOH or TAM. (A) Scatter plot showing liver mass normalized to total body mass, ±s.d. *P*-values derived from Kruskal–Wallis non-parametric ANOVA followed by Dunn's multiple comparisons test. (B) Stacked bar graph showing the percentage of zebrafish per tested group categorized as no/minimal changes, intermediate changes, or HCC. *P*-values derived using Fisher's exact test comparing samples with and without HCC. (C) Representative H&E stained histological images from TAM-treated zebrafish. Left panels: control liver showing normal architecture with scattered bile ducts (arrowhead, top panel) and round, smooth, similarly sized nuclei (bottom panel); it was scored as no/minimal changes. Middle panels: CreLox liver showing minimal architectural abnormalities (top panel) and mild cytologic abnormalities including focal nuclear enlargement (arrowhead, bottom panel); it was scored as intermediate changes. Right panels: CreLox liver with moderate architectural abnormalities including pseudoglands (arrowhead, top panel) and moderate cytologic abnormalities including enlarged irregularly shaped nuclei (arrowheads, bottom panel); it was scored as HCC. Scale bars: 20 µm (top panels) and 10 µm (bottom panels).

To determine HCC incidence at 6 mpf, we determined liver-to-body-mass ratios and performed histologic analysis of male and female zebrafish. Hematoxylin and Eosin-stained slides were blinded and examined by a board-certified pathologist with subspecialty fellowship training in liver pathology (K.J.E.), and HCC was diagnosed in accordance with previously defined criteria (Evason et al., 2015; Mudbhary et al., 2014). In addition to categorizing samples as ‘HCC’ or ‘no/minimal changes’, we also included an ‘intermediate’ category to account for samples showing abnormalities that did not meet diagnostic criteria for HCC. As we reported previously, *Tg(fabp10a:pt-β-cat)* zebrafish exhibited significantly increased liver-to-body mass ratios (*P*<0.0001) and had significantly more HCC by histologic analysis than non-transgenic control siblings (85% versus 0%; *P*=0.0002) (Fig. 3A–C).

TAM-treated CreLox zebrafish exhibited significantly increased liver-to-body mass ratios (*P*=0.0032) and had significantly more HCC histologically than TAM-treated control siblings (27% versus 0%; *P*=0.0006) (Fig. 3A–C). TAM-treated CreLox zebrafish also displayed intermediate changes more frequently than TAM-treated sibling controls (33% versus 5%; *P*=0.0029) (Fig. 3B,C). These results indicate that CreERT2-mediated switching on of activated β-catenin in most hepatocytes, beginning at 3 dpf, leads to HCC in adult zebrafish.

In the absence of TAM, CreLox zebrafish exhibited slightly increased liver-to-body mass ratios compared to sibling controls, although this difference was not statistically significant (*P*=0.0569). Liver-to-body mass ratios in CreLox zebrafish were similar in the presence and absence of TAM (*P*=0.1559). We found that 13% of vehicle-treated CreLox zebrafish showed HCC by histologic analysis, significantly more than in vehicle-treated control siblings (0%; *P*=0.0460) and similar to TAM-treated CreLox zebrafish (27%; *P*=0.3334) (Fig. 3B,C). The rate of intermediate changes in vehicle-treated CreLox zebrafish was also significantly greater than in vehicle-treated control siblings (43% versus 3%; *P*=0.0001) and similar to the rate in TAM-treated CreLox siblings (*P*=0.5959) (Fig. 3B,C). The finding of HCC and intermediate changes in CreLox zebrafish in the absence of TAM supports the hypothesis that switching on activated β-catenin in a subset of larval hepatocytes is sufficient to drive liver tumorigenesis.

Taken together, these results show that TAM treatment in CreLox zebrafish, which leads to switching on β-catenin in most hepatocytes beginning at 3 dpf, did not significantly increase HCC burden compared to vehicle treatment. The finding that the number of hepatocytes switched on from 3 dpf onward does not significantly affect hepatocarcinogenesis suggests that the critical time point for switching on β-catenin to initiate HCC occurs at or before 3 dpf. This finding also implies that switching occurs before 3 dpf in *Tg(fabp10a:CreERT2);Tg(ubi:switch)* zebrafish as well. The absence of significant switching by immunofluorescence analysis of *Tg(fabp10a:CreERT2);Tg(ubi:switch)* larvae at 6 dpf (Fig. 2A,B and Fig. S1) could be due to limitations in our detection and analysis strategy and/or the time required for degradation of EGFP.

### TAM treatment of adult zebrafish does not enhance HCC formation

**Fig. 4.**
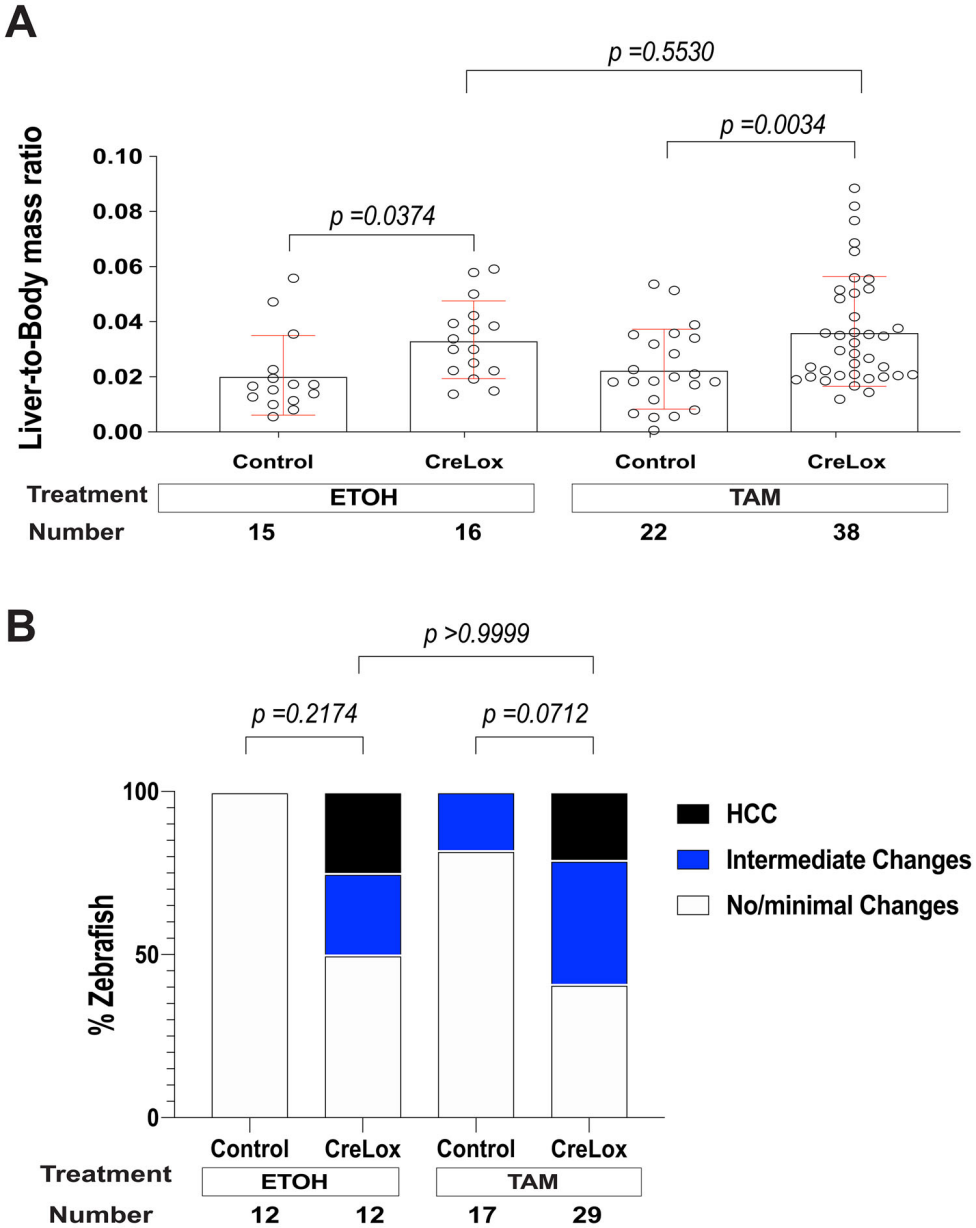
**Switching on additional activated β-catenin in adult zebrafish does not increase HCC penetrance.** CreLox [*Tg(fabp10a:CreER^T2^); Tg(fabp10a:flox-pt-β-cat)*] zebrafish and control siblings lacking either the Cre driver or lox-switch transgene [*Tg(fabp10a:flox-pt-β-cat)* and *Tg(fabp10a:CreER^T2^)*, control], were treated with 4-hydroxytamoxifen (TAM) or vehicle (ethanol, EtOH) at 3 mpf. Livers were weighed and examined microscopically 6 months later. (A) Scatter plot showing liver mass normalized to total body mass, ±s.d. *P*-values were obtained using ordinary one-way ANOVA followed by Sidak's multiple comparisons test. Data from four experiments were pooled. (B) Stacked bar graph showing the percentage of zebrafish per tested group categorized as no/minimal changes, intermediate changes, or HCC. *P*-values derived using Fisher's exact test, comparing samples with and without HCC. Data from three experiments were pooled*.*

The above analysis of the CreLox model demonstrates that switching on activated β-catenin in a small subset of early larval hepatocytes is sufficient to initiate HCC. To test the hypothesis that switching on activated β-catenin in a subset of hepatocytes in adult zebrafish can initiate HCC, we assessed TAM- or vehicle-treated 3-mpf adult male and female CreLox zebrafish and control siblings lacking either the Cre driver or the lox-switch transgene. Zebrafish livers were analyzed 6 months post switching at 9 mpf with respect to liver size and histology. TAM-treated CreLox zebrafish exhibited increased liver-to-body mass ratios compared to TAM-treated control siblings (*P*=0.0034) (Fig. 4A). Vehicle-treated CreLox zebrafish also exhibited liver-to-body mass ratios that were significantly greater than vehicle-treated control siblings (*P*=0.0374) and similar to TAM-treated CreLox siblings (*P*=0.5530) (Fig. 4A). With either TAM or vehicle treatment, CreLox zebrafish showed HCC histologically (21% versus 25%, *P*>0.9999) (Fig. 4B). Additionally, the rate of intermediate changes was similar in CreLox zebrafish with TAM (38%) or vehicle (25%) treatment (*P*=0.4936) (Fig. 4B). The similar HCC incidence in CreLox zebrafish with and without adult TAM treatment indicates that switching on activated β-catenin in additional cells (25% of hepatocytes, Fig. 2F) during adulthood does not enhance HCC formation. These results support the hypothesis that switching on β-catenin during development is necessary for hepatocarcinogenesis.

Independent of TAM treatment, 22% of CreLox zebrafish had HCC at 9 mpf, significantly more than control siblings lacking either the Cre driver or the lox-switch transgene (*P*=0.0082, Fisher's exact test). This finding provides additional support for the hypothesis that switching on β-catenin in a small number of early larval hepatocytes is sufficient to initiate HCC.

### Wnt reporter activity is heterogeneous in zebrafish HCC

Having demonstrated that switching on activated β-catenin in a subset of larval hepatocytes is sufficient to initiate HCC, we next wanted to test the related hypothesis that β-catenin-dependent gene transcription is heterogeneous during HCC progression. We compared Wnt pathway activity in adult male and female *Tg(fabp10a:pt-β-cat)* zebrafish HCC (Evason et al., 2015) and non-transgenic control sibling livers at 6 mpf by crossing these zebrafish to the Wnt reporter line *Tg(7xTCF-Xla.Siam: mCherry)* (Moro et al., 2012). In parallel, we performed immunofluorescence staining for β-catenin on the same liver cryosections as well as on separate paraffin-embedded samples from *Tg(fabp10a:pt-β-cat)*, CreLox, and control sibling livers.

**Fig. 5.**
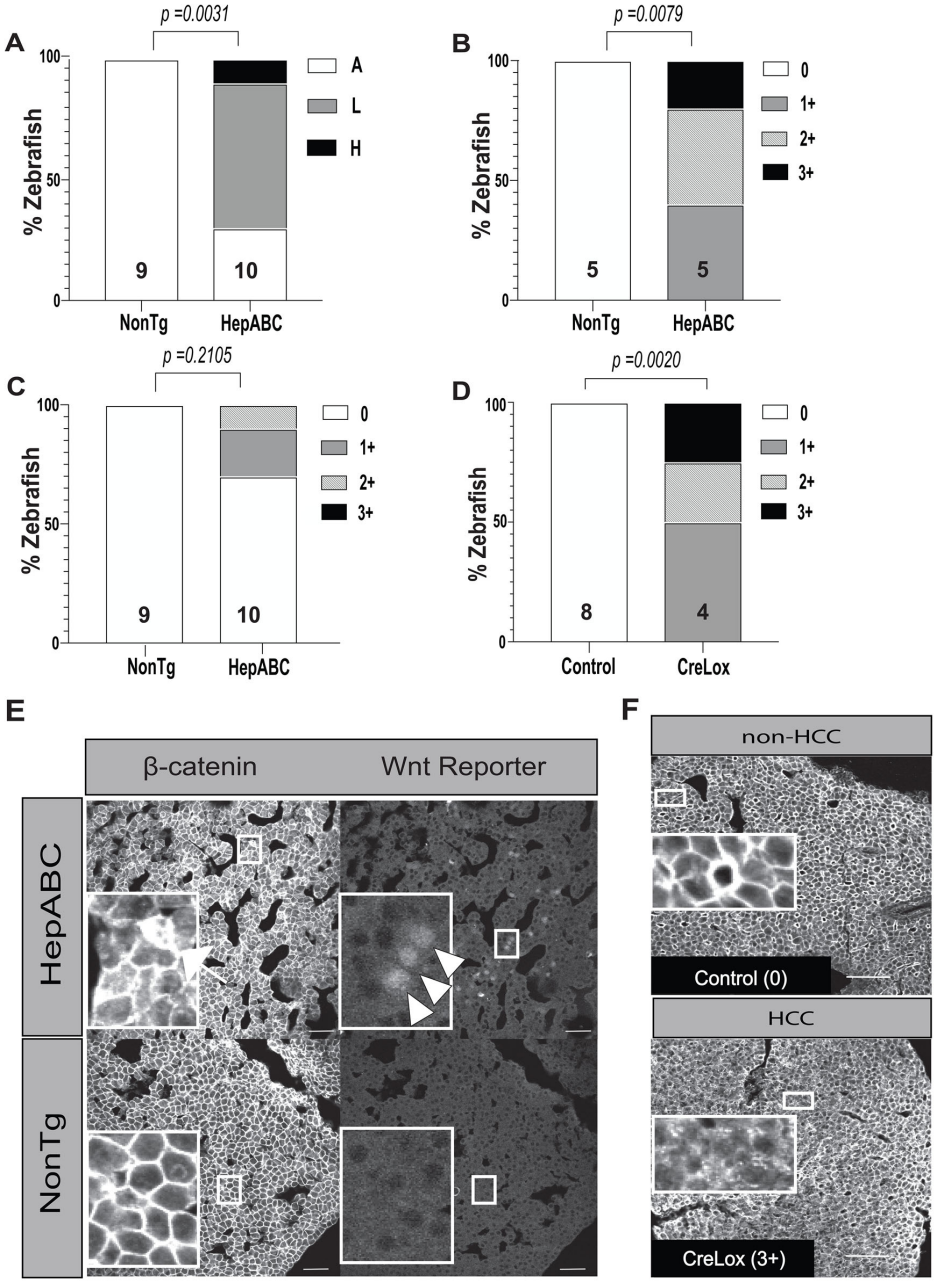
**Heterogeneous Wnt reporter expression and β-catenin cytoplasmic localization in adult β-catenin-driven HCC.** (A) Stacked bar graph showing analysis of Wnt reporter (*7xTCF-Xla.Siam:*mCherry) expression in liver tissue of *Tg(fabp10a:flox-pt*-*β-cat)* (HepABC) HCC and zebrafish livers of sibling controls lacking this transgene (NonTg). Wnt reporter expression was scored as: A, absent (no mCherry expression); L, low (mCherry expression in less than 10% of cells); or H, high (mCherry expression in greater than 10% of cells). *P*-values derived from Fisher's exact test comparing samples with (low or high) and without (absent) Wnt reporter expression. Graph shows data from one experiment. (B,C) Stacked bar graphs showing quantification of β-catenin localization by immunofluorescence staining performed on paraffin-embedded sections (B) or cryosections (C) in HepABC HCC and NonTg zebrafish livers. Samples were scored based on amount of cytoplasmic staining: 0, no cytoplasmic staining; 1+, focal (<10%) weak to moderate cytoplasmic staining; 2+, focal strong cytoplasmic staining or patchy (10–50%) weak to moderate cytoplasmic staining; 3+, diffuse (>50%) cytoplasmic staining. *P*-values derived from Fisher's exact test comparing samples with membrane staining only (0+) to those with cytoplasmic staining (1+ to 3+). Each experiment was performed once. (D) Stacked bar graph showing quantification of β-catenin localization in 6-mpf CreLox zebrafish with HCC and control siblings without HCC lacking either the Cre driver or lox-switch transgene [*Tg(fabp10a:flox-pt-β-cat)* and *Tg(fabp10a:CreER^T2^)*, control]*.* Numbers above x axis indicate the sample size for each group. *P*-values determined by Fisher's exact test. Experiment was performed once. (E) Representative β-catenin and Wnt reporter images of cryosections from a HepABC liver diagnosed as HCC (top panels) and a NonTg liver diagnosed as no/minimal changes (bottom panels). Arrow indicates a cell with cytoplasmic β-catenin localization; white arrowheads indicate Wnt reporter expression. Scale bars: 30 µm. Insets contain 5× magnified images of regions of tissue in smaller boxes for each image. (F) Representative immunofluorescence images of β-catenin staining. Control zebrafish showed membrane staining only, whereas CreLox zebrafish with HCC showed varying degrees of cytoplasmic staining. Scale bars: 50 µm. In each image, large inset box is 5× magnification of small box.

We found that 70% of *Tg(fabp10a:pt-β-cat)* zebrafish HCC showed Wnt reporter expression, which was mostly heterogeneous, involving less than 10% of cells in 60% of HCCs and greater than 10% of cells in 10% of HCCs (Fig. 5A,D). No Wnt reporter activity was seen in hepatocytes from non-transgenic sibling controls, none of which had HCC (*P*=0.0031, Fisher's exact test). As we reported previously, all non-transgenic non-HCC zebrafish livers exhibited only membrane localization of β-catenin (Fig. 5B,C,E). In contrast, most HCC (53% total; 100% of cryosections and 30% of paraffin-embedded sections) from *Tg(fabp10a:pt-β-cat)* zebrafish showed at least some cytoplasmic β-catenin staining (Fig. 5B,C,E), consistent with our previous results (Evason et al., 2015). Cytoplasmic β-catenin localization was heterogeneously distributed within HCC tissue: 27% of zebrafish showed weak to moderate cytoplasmic staining in less than 10% of hepatocytes; 20% showed strong cytoplasmic staining in less than 10% of hepatocytes or weak to moderate cytoplasmic staining in 10–50% of hepatocytes; and 7% showed cytoplasmic staining in greater than 50% of hepatocytes (Fig. 5B,C,E).

We noted similar findings in HCC from CreLox zebrafish: 50% of zebrafish showed cytoplasmic β-catenin in less than 10% of hepatocytes, 25% showed cytoplasmic β-catenin in 10–50% of hepatocytes, and 25% showed cytoplasmic β-catenin in greater than 50% of hepatocytes (Fig. 5D,F). All non-HCC zebrafish livers from control siblings lacking the Cre driver or lox-switch transgene showed only membrane localization of β-catenin (*P*=0.0020, Fisher's exact test) (Fig. 5D,F). These results indicate that whether activated β-catenin is switched on diffusely [*Tg(fabp10a:pt-β-cat)*] or in a subset of hepatocytes (CreLox) during early development, as adults these zebrafish develop HCC showing a similar heterogeneous pattern of β-catenin localization. Together, these experiments show that β-catenin-driven transcriptional activity is heterogeneous in zebrafish β-catenin-driven HCC, implying that the heterogeneous nuclear and cytoplasmic β-catenin staining observed in these tumors is not due to limitations in detection.

### Gene expression analysis of β-catenin-driven zebrafish HCC

We found that HCC incidence in the CreLox zebrafish model was relatively low (13% to 27% at 6 mpf, Fig. 3B) compared to that in *Tg(fabp10a:pt-β-cat)* zebrafish (85% at 6 mpf, Fig. 3B) (Evason et al., 2015). This low HCC incidence provided an opportunity to examine transcriptional changes in CreLox zebrafish without HCC, yielding insights into mechanisms of hepatocarcinogenesis. We performed RNA sequencing (RNA-seq) on livers with and without HCC from adult male CreLox zebrafish. As controls, we performed RNA-seq on livers without HCC from male siblings with the Cre driver transgene only.

**Fig. 6.**
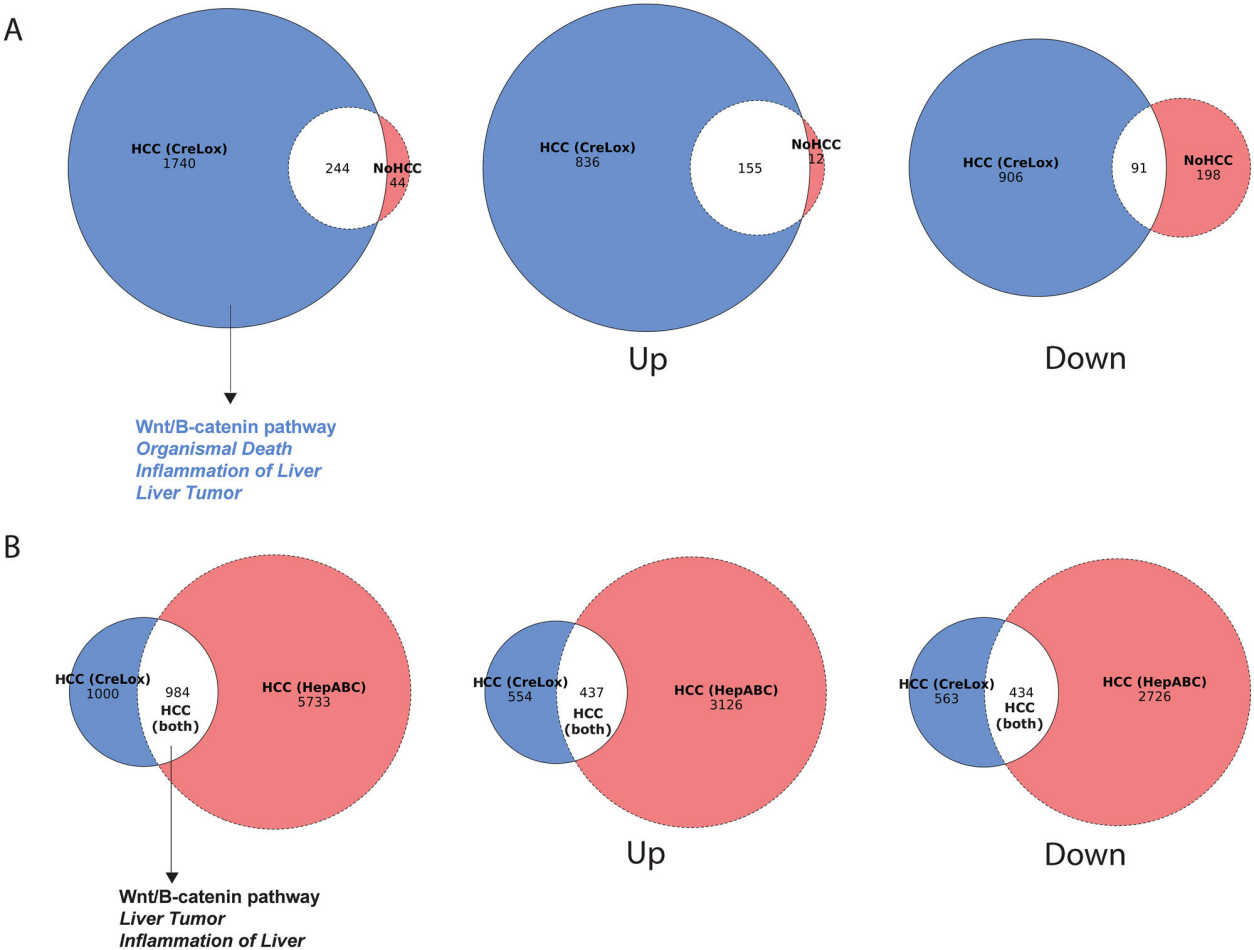
**Bulk RNA sequencing demonstrates similarities in β-catenin-driven HCC models.** (A) Venn diagrams showing the overlap of genes significantly dysregulated in livers from CreLox zebrafish with HCC and sibling CreLox zebrafish with no HCC. Overlap in all genes (left panel), upregulated genes (middle panel), and downregulated genes (right panel) are shown. Left panel also shows pathways unique to CreLox zebrafish with HCC, as determined by Ingenuity Pathway Analysis. Regular text indicates pathways curated under Canonical Pathways and italicized text indicates pathways curated under Diseases and Functions. (B) Venn diagrams showing the overlap of genes significantly dysregulated in CreLox HCC and HepABC HCC. Overlap in all genes (left panel), upregulated genes (middle panel), and downregulated genes (right panel) are shown. Left panel also shows pathways shared between CreLox HCC and HepABC HCC, as determined by Ingenuity Pathway Analysis. Regular text indicates pathways curated under Canonical Pathways and italicized text indicates pathways curated under Diseases and Functions.

We identified 1984 significantly dysregulated genes in CreLox zebrafish with HCC and 288 significantly dysregulated genes in CreLox zebrafish without HCC compared to sibling controls [Fig. 6A, Gene Expression Omnibus (GEO) accession number GSE137788]. Ingenuity Pathway Analysis (IPA) (Ingenuity Systems, www.ingenuity.com) of differentially expressed genes revealed that cancer was the most significantly affected ‘Diseases and Disorders’ in CreLox zebrafish HCC (Table S2), indicating cancer-related pathways are significantly altered in this model. We found that 1752 (88%) of dysregulated transcripts in CreLox zebrafish with HCC were not dysregulated in CreLox zebrafish without HCC (Fig. 6A, Tables S3–S5). These data indicate genes and pathways that are HCC-specific and may be important in driving hepatocarcinogenesis.

We next tested the hypothesis that HCCs initiated by switching on activated β-catenin in a subset of hepatocytes (CreLox model) or ubiquitously in hepatocytes [*Tg(fabp10a:pt-β-cat*) model] are transcriptionally similar. We performed RNA-seq on adult male *Tg(fabp10a:pt-β-cat*) zebrafish HCC and non-transgenic sibling controls and identified 6717 significantly dysregulated genes (Fig. 6B, GEO accession number GSE137788). We found that 989 (50%) of dysregulated transcripts in CreLox HCC were shared with *Tg(fabp10a:pt-β-cat)* HCC (*P*=6.3e-121; Fisher's exact test) (Fig. 6B, Tables S6–S8) (Shen). This analysis indicates that there are significant transcriptional similarities in β-catenin-driven HCC regardless of the number of cells in which the activated β-catenin transgene is present and active during HCC initiation.

### Single-cell RNA sequencing of β-catenin-driven zebrafish HCC

**Fig. 7.**
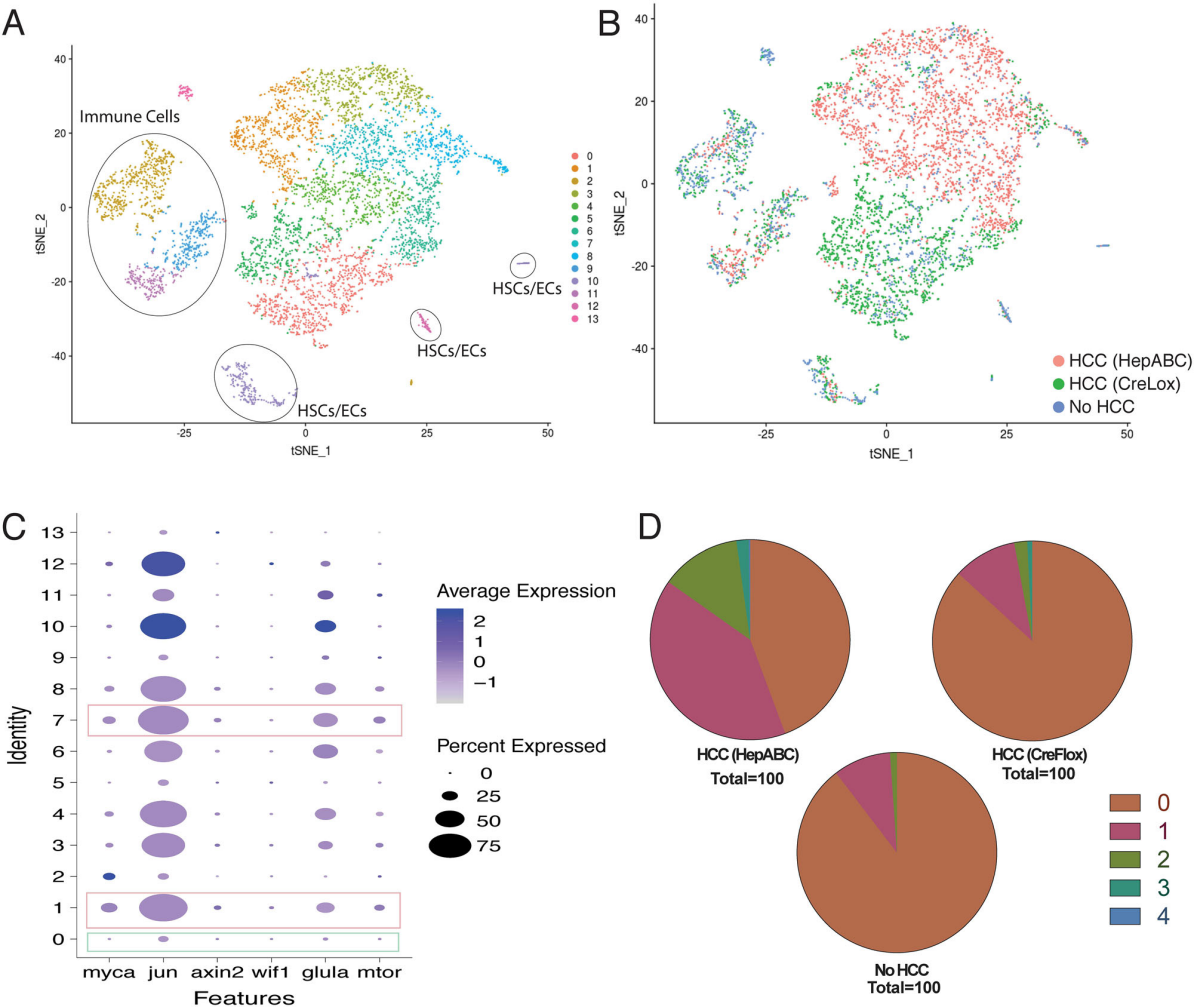
**Single-cell RNA sequencing shows heterogeneous transcriptional profiles in β-catenin-driven HCC.** (A) t-SNE plot of cells from all three samples [HCC from CreLox and *Tg(fabp10a:pt-β-cat)* zebrafish and non-HCC control liver] following multi-sample integration, color-coded by their associated cluster (0–13). Cell types of each cluster were determined based on differential expression of genes highlighted in Table S22: hepatocytes (non-circled cells), clusters 0, 1, 3, 4, 5, 6, 7, 8; immune cells, clusters 2, 9 and 11; hepatic stellate cells/endothelial cells, clusters 10 and 12; erythrocytes, cluster 13. (B) tSNE plot of multi-sample integration of cells from all three samples, color-coded by their sample of origin: *Tg(fabp10a:pt-β-cat)* (HepABC) HCC, pink; CreLox HCC, green; and no HCC control, blue. (C) Dot plot of β-catenin target genes differentially expressed across clusters 0–13 from A. Dot size represents the percentage of cells within the cluster that contribute to expression, and color intensity represents the average normalized level of gene expression. Pink rectangles highlight clusters with the lowest relative number of hepatocytes from non-HCC liver (clusters 1 and 7, Table S9), and green rectangles highlight clusters with the highest relative number of hepatocytes from non-HCC liver (cluster 0, Table S9). (D) Pie graphs showing the percent of hepatocytes in each liver (Table S16) that expressed 0 (brown), 1 (pink), 2 (green), 3 (teal), or 4 (blue) of Wnt/β-catenin target genes *axin2*, *mtor*, *glula*, *myca* and *wif1*.

To transcriptionally characterize β-catenin-driven zebrafish HCC at the single-cell level, we performed single-cell RNA sequencing of 1 liver each from the following adult male zebrafish: (1) CreLox zebrafish with HCC; (2) *Tg(fabp10a:pt-β-cat)* zebrafish with HCC; and (3) zebrafish with no HCC containing the lox-switch transgene only (control) (Fig. S4). We combined the data from all three samples and performed an integrated linear-dimensional reduction analysis, following the t-Distributed Stochastic Neighbor Embedding (t-SNE) tSNE algorithm, to visualize transcriptional distances between cells irrespective of their sample of origin. For the most part, cells clustered based on cellular identity as opposed to sample of origin (Fig. 7A,B). For example, hepatic stellate cells (HSCs) and endothelial cells (ECs) from all three samples are located in cluster 10 (Fig. 7A). To a large extent, hepatocytes from CreLox HCC liver clustered separately from hepatocytes of *Tg(fabp10a:pt-β-cat)* HCC (Fig. 7B). Genes significantly upregulated in cluster 5, predominantly consisting of hepatocytes from CreLox HCC (Table S9), included those involved in oxidation reduction, isoprenoid biosynthesis, lipoprotein metabolism and liver development (Tables S10–S11). Genes significantly upregulated in clusters 4 and 7, predominantly consisting of hepatocytes from *Tg(fabp10a:pt-β-cat)* HCC (Table S9), included those involved in endopeptidase inhibitor activity, serine-type endopeptidase inhibitor activity, cysteine-type endopeptidase inhibitor activity and protein binding (Tables S12–S15). These data show that on a cell-by-cell basis, there was substantial intertumor heterogeneity in these two β-catenin-driven HCC samples.

We examined the level and distribution of selected Wnt/β-catenin target genes across hepatocyte and non-hepatocyte clusters, focusing on genes that were significantly upregulated in male zebrafish HCC from our bulk RNA-seq analysis (*jun*, *axin2*, *wif1*, *myca*) (Table S6) and/or previously implicated in hepatocarcinogenesis (*jun*, *axin2*, *mtor*, *glula*, *myca*) (Adebayo Michael et al., 2019; Okabe et al., 2016; Seki et al., 2012; Villanueva et al., 2008). We observed non-uniform expression levels and distribution of these genes across clusters (Fig. 7C), indicating heterogeneity in Wnt/β-catenin target gene expression. We determined that 2.9% (CreLox HCC) to 15.2% [*Tg(fabp10a:pt-β-cat)* HCC] of hepatocytes from zebrafish β-catenin-driven HCC had detectable expression of two or more of the Wnt/β-catenin target genes *axin2*, *mtor*, *glula*, *myca* and *wif1* (Fig. 7D and Table S16), and 0.8% (CreLox HCC) to 2.2% [*Tg(fabp10a:pt-β-cat)* HCC] of hepatocytes had detectable expression of three or more of these genes. One Wnt/β-catenin target, *jun*, was widely expressed in non-HCC hepatocytes (52.6%), suggesting that the presence of *jun* mRNA is not indicative of aberrant Wnt/β-catenin signaling (Table S17). The level of *jun* expression in non-HCC hepatocytes expressing *jun* was significantly lower than in *jun*-expressing *Tg(fabp10a:pt-β-cat)* HCC and CreLox HCC hepatocytes (*P*=0.0004 and *P*<0.0001, respectively) (Table S17). This analysis of Wnt/β-catenin target gene expression at the single-cell level indicates focal activation of Wnt/β-catenin signaling in established tumors, supporting the hypothesis that β-catenin-driven transcriptional activity is heterogeneous in zebrafish β-catenin-driven HCC.

## DISCUSSION

Here we report generation of a zebrafish model of β-catenin-driven HCC [*Tg(fabp10a:CreERT2)*; *Tg(fabp10a:flox-pt-β-cat),* CreLox], in which activated β-catenin is switched on using tamoxifen-inducible CreER^T2^ recombinase under control of the hepatocyte-specific *fabp10a* promoter. This tool enables switching on activated β-catenin in a subset (∼9%) of larval hepatocytes or in the majority of larval hepatocytes with 4-hydroxytamoxifen (TAM) treatment. Switching rates were estimated using the *Tg(ubi:switch)* reporter line to visualize both pre- and post-switch states with fluorescent proteins. We discovered that switching on activated β-catenin in the majority of hepatocytes by TAM administration from 3 to 6 dpf was sufficient to produce HCC in 28% of zebrafish by 6 mpf. Switching on activated β-catenin in a subset of larval hepatocytes led to similar HCC penetrance, indicating that activated β-catenin in a small number of developing hepatocytes is sufficient to initiate HCC and suggesting that the critical time window for turning on activated β-catenin occurs before 3 dpf.

HCC penetrance was significantly lower in the CreLox model than in *Tg(fabp10a:pt-β-cat)* zebrafish (Evason et al., 2015), in which activated β-catenin is expressed under the direct control of the *fabp10a* promoter beginning at 2 dpf (Her et al., 2003a). One possible explanation for this difference in penetrance is that activated β-catenin is expressed slightly earlier in *Tg(fabp10a:pt-β-cat)* zebrafish than in CreLox zebrafish due to the time required to transcribe and translate *CreER^T2^* and excise the BFP cassette. Perhaps a specific subset of larval *fabp10a-*expressing hepatocytes is ‘oncogene sensitive’ and must express activated β-catenin at an early time point in order to initiate tumorigenesis. In CreLox zebrafish, HCC only develops when activated β-catenin happens to be switched on in a sufficient number of these sensitive hepatocytes. In contrast, *Tg(fabp10a:pt-β-cat)* zebrafish have activated β-catenin in all *fabp10a-*expressing hepatocytes, including the oncogene-sensitive hepatocytes, and thus develop HCC at a higher rate.

Activated β-catenin expression in a progenitor population representing 4% of liver cells at E11.5 is sufficient to initiate HCC in mice (Mokkapati et al., 2014). Taken together with our findings in zebrafish, these results support the hypothesis that β-catenin expression in a subset of hepatocytes is sufficient to initiate HCC in vertebrates. HCC initiation may require the relatively undifferentiated, primitive nature of these susceptible cells along with β-catenin. In mice, the initiating cells are multipotent and express hepatic stem cell markers (Mokkapati et al., 2014). In zebrafish, early liver cells have exceptional regenerative potential, as zebrafish liver structure and function normalize within 4 days following severe hepatocyte ablation (Choi et al., 2014). In HCC patients, the inflammatory, regenerative milieu of chronic hepatitis – present in 80–90% of HCC – may provide the analogous additional impetus for hepatocarcinogenesis (Fattovich et al., 2004).

In adult zebrafish with β-catenin-driven HCC, we found that Wnt reporter activity (Fig. 5A,E) and Wnt target gene expression (Fig. 7C,D) are confined to a small subset of HCC cells. These findings are in keeping with the focal nuclear/cytoplasmic β-catenin localization that we observed in both of our β-catenin-driven HCC models (Fig. 5B–F) (Evason et al., 2015) and that is characteristic of human HCC (Evason et al., 2015; Friemel et al., 2015; Qiao et al., 2018; Rebouissou et al., 2016). Our results support the hypothesis that heterogeneity in β-catenin immunostaining is due to heterogeneous β-catenin activity and not due to limitations in detection of β-catenin protein.

To switch on activated β-catenin, we used *CreER^T2^*, consisting of Cre recombinase fused to the modified ligand-binding domain of the human estrogen receptor (ER^T2^). CreER^T2^ is believed to be the best tamoxifen-dependent Cre recombinase in terms of ligand specificity – ER^T2^ binding is specific for 4-hydroxytamoxifen (TAM), limiting the risk of activation of fusion proteins by endogenous estrogens – and recombination efficiency (Feil et al., 1997; Metzger and Chambon, 2001). We found that ∼9% of larval hepatocytes in *Tg(fabp10a:CreERT2)*; *Tg(ubi:switch)* zebrafish showed switching in the absence of TAM, indicating leaky CreER^T2^ activity in this model. A study by Choi et al. used the same *fabp10a* promoter that we used in our studies to drive *CreER^T2^* expression in zebrafish and did not report leakiness, although adult switching was not closely examined (Choi et al., 2014). However, mouse studies have demonstrated tamoxifen-independent CreER^T2^ activity in some circumstances, including when CreER^T2^ is expressed under control of the Ubiquitin C promoter (Kristianto et al., 2017) and in the RIP-CreER mouse, wherein pancreatic β cells are targeted with the rat insulin 2 promoter (Liu et al., 2010). These observations and the findings reported here highlight the importance of maintaining proper controls to avoid confounding effects of TAM-independent CreER^T2^ activity.

Our finding that TAM treatment of adult CreLox zebrafish did not increase HCC penetrance suggests that additional activated β-catenin in adult zebrafish is not sufficient to initiate HCC. This finding is in keeping with results in mice showing that hepatocyte-specific expression of β-catenin in mice via hydrodynamic tail vein injection (Tao et al., 2016) or under control of the albumin promoter (Nejak-Bowen et al., 2010) is not sufficient for HCC initiation. However, to more rigorously examine the question of timing, tighter control of β-catenin expression will be required. This objective could be achieved by modifying the *fabp10a* promoter to reduce CreER^T2^ expression levels (Morita et al., 2012). A doxycycline-inducible system (Li et al., 2012) could enable determining the effects of turning on and off β-catenin at different time points.

Graph-based clustering of our scRNA-seq data showed some integration of hepatocytes derived from the two HCC models, but several hepatocyte clusters, including clusters 4, 5 and 7, were markedly enriched for one HCC model over the other (Fig. 7A,B and Table S9). There are at least three possible explanations for this finding. One, technical issues in sample preparation might cause sample-specific effects to dominate over cell-type specific effects. In this case, however, we would expect to see separation of both non-hepatocytes and hepatocytes based on sample type, and in contrast we saw clear integration of all three samples in clusters 9, 10 and 11 (Fig. 7A,B). Two, CreLox and *Tg(fabp10a:pt-β-cat)* zebrafish may develop distinct types of HCC. Repudiating this possibility, histologic analysis of CreLox and *Tg(fabp10a:pt-β-cat)* zebrafish does not reveal substantial morphologic differences in HCC derived from the two models. Three, HCC in CreLox and *Tg(fabp10a:pt-β-cat)* may be similar overall, but substantial intertumor heterogeneity among different animals results in separation of clusters from any two animals regardless of model type. Supporting the third possibility, our bulk RNA-seq analysis highlighted significant similarities in gene expression and pathway alterations between CreLox and *Tg(fabp10a:pt-β-cat)* zebrafish HCC. We also saw similar heterogeneous β-catenin localization in both HCC models. scRNA-seq analysis of additional tumors from both models would be helpful to further define similarities and differences among β-catenin-driven HCC. It will also be interesting to see if HCCs in these models consist of a single large tumor or multiple tumors. This will clarify the context in which heterogenous β-catenin arises. Tracking tumor cell origin using tools such as CRISPR/Cas9-mediated genome editing of synthetic target arrays for lineage tracing can provide these answers (McKenna et al., 2016).

In summary, we have generated and characterized a CreER-inducible zebrafish model of hepatocyte-specific β-catenin-driven HCC. We have defined the transcriptional characteristics of β-catenin-driven zebrafish HCC at both the bulk and single-cell transcriptome levels. Our findings underscore the importance of a small number of β-catenin-expressing cells in driving both HCC initiation and progression.

## MATERIALS AND METHODS

### Zebrafish husbandry

Zebrafish (*Danio rerio*) lines were maintained under standard conditions in compliance with the University of Utah Institutional Animal Care and Use Committee guidelines (Kimmel et al., 1995). In addition to male and female wild-type AB and TL strains, six transgenic lines were used in this study: *Tg(fabp10a*:*pt-β-cat)* (Evason et al., 2015)*, Tg(7xTCF-Xla.Siam:nlsmCherry)* (Moro et al., 2012), *Tg(ubi:loxP-EGFP-loxP-mCherry)* [referred to hereafter as *Tg(ubi:switch)*] (Mosimann et al., 2011)*, Tg(fabp10a:Cre)* (Ni et al., 2012), *Tg(fabp10a: CreER^T2^),* and *Tg(fabp10a:flox-pt-β-cat).* Embryos and larvae were cultured in egg water (2.33 g Instant Ocean in 1 l Milli-Q water with 0.5 ml Methylene Blue) and stored in a 28.5°C incubator. Adult zebrafish were housed on a recirculating system and fed brine shrimp, flakes, and powdered food. Animals were euthanized by tricaine methanesulfonate (0.02%) and/or ice water immersion (rapid chilling).

### Generation of *Tg(fabp10a: CreER^T2^)* and *Tg(fabp10a:loxP-BFP-loxP-Xla.Ctnnb1)* zebrafish

The *pKE13_fabp10a:CreER^T2^,cryaa:Venus* plasmid was generated by replacing the *Cre* portion of the *I-SceI* meganuclease vector *pHD157_fabp10a:Cre,cryaa:Venus* (a kind gift from Dan Hesselson of the Garvan Institute of Medical Research) with tamoxifen-inducible *CreER^T2^* (Ninov et al., 2013). The *CreER^T2^* insert (Ninov et al., 2013) was amplified by polymerase chain reaction (PCR) from *TP1:CreERT2-Tol2-delta* (a kind gift from Nikolay Ninov of the Center for Regenerative Therapies TU Dresden), adding NheI and NotI restriction enzyme sites at the 5′ and 3′ ends, respectively, and placed downstream of the *fabp10a* promoter (Her et al., 2003b) into an *I-SceI* meganuclease vector (Thermes et al., 2002) that also contained *cryaa:Venus* (Hesselson et al., 2009; Kurita et al., 2003).

The *pKE12_fabp10a:loxP-BFP-loxP-Xla.Ctnnb1,cryaa:mCherry* plasmid was generated from the *I-SceI* meganuclease vector *pHD142_ins:loxP-BFP-loxP-DTA,cryaa:mCherry* (a kind gift from Dan Hesselson) by replacing the insulin promoter and diphtheria toxin portions with the *fabp10a* promoter (Her et al., 2003b) and *pt-β-catenin*, respectively. *pt-β-catenin* was amplified by PCR from the pCS2-XE49 plasmid (Yost et al., 1996) (a kind gift from Ben Cheyette of University of California, San Francisco), adding FseI and NotI sites at the 5′ and 3′ ends, respectively, and placed downstream of the *fabp10a* promoter (Her et al., 2003b) and floxed enhanced blue fluorescent protein (EBFP2) into an *I-SceI* meganuclease vector (Thermes et al., 2002) that also contained *cryaa:mCherry* (Hesselson et al., 2009; Kurita et al., 2003).

One-cell-stage embryos were microinjected with *pKE12* or *pKE13* plasmid, *I-SceI* meganuclease, *I-SceI* buffer, and Phenol Red as previously described (Thermes et al., 2002). Injected embryos with colored eyes at 2–5 days post fertilization (dpf) were raised to adulthood and crossed to detect founders with germline transmission. We identified two *Tg(fabp10a:CreER^T2^)* founders, showing similar phenotypes, and one *Tg(fabp10a:loxP-BFP-loxP-Xla.Ctnnb1)* founder [hereafter referred to as *Tg(fabp10a:flox-pt-β-cat)*]. Transgenic descendants of *Tg(fabp10a:CreER^T2^)* and *Tg(fabp10a:flox-pt-β-cat)* founders were differentiated from control siblings at 3 dpf or later based on *cryaa:*Venus and *cryaa:*mCherry expression, respectively. For experiments where it was not possible to distinguish *cryaa:*Venus expression due to the presence of *Ubi:*GFP, *Tg(fabp10a:CreER^T2^)* animals were identified by PCR genotyping (see below).

### PCR genotyping of *Tg(fabp10a:CreER^T2^)*

For genotyping of larvae or adult fish, genomic DNA was extracted by immersing whole larvae or fin-clip in 100 μl of genomic extraction buffer with 25 μl Proteinase K per ml of genomic extraction buffer. Samples were cycled as follows: 55°C 120′, 95°C 10′ and 10°C hold. 1 μl of this liquid was used in a 12 μl total volume PCR reaction. For amplifying CreER, 5′-AACGAGTGATGAGGTTCGCA–3′ forward primer and 5′–TGATCCTGGCAATTTCGGCT–3′ reverse primer were used. Temperature cycles were as follows: 95°C for 2′; 40 cycles of 95°C for 30″, 54°C for 30″, 72°C for 2′30″; 72°C for 5′; and 10°C hold. PCR samples were loaded into a 3% agarose gel in PBS and a band size of 488 bp was observed to establish Cre positivity. For Cre genotyping from frozen or paraffin-embedded sections, we used zebrafish material from the embedded blocks as input for genomic DNA extraction.

### 4-hydroxytamoxifen (TAM) treatment

At 3 dpf, hatched larvae were anesthetized in tricaine methanesulfonate for fluorescence sorting based on fluorophore expression in the eyes. Sorted larvae were distributed into nine- or six-well culture plates in 3 or 4 ml solution of egg water with either 10 µM 4-hydroxytamoxifen (TAM, Sigma-Aldrich) and 0.1% ethanol or 0.1% ethanol alone from 3 to 6 dpf. A maximum of 5 larvae/ml solution were treated. Untreated larvae were kept in egg water only with no additions.

Adult zebrafish (3–4 mpf) were immersed in system water alone; system water plus 0.1% ethanol; or system water plus 0.1% ethanol and 2.3 µM TAM overnight for 3 consecutive nights. During the day, fish were fed and exposed to normal water flow. The fish were euthanized by rapid freezing either 1 week post treatment for assessing switching or 6 months post treatment for assessing HCC induction.

### Whole-mount immunostaining and imaging of larval zebrafish

Larvae were euthanized, rinsed in PBS, and then fixed in 4% paraformaldehyde (PFA) in PBS for at least 12 h at 4°C. Post-fixation, larvae were rinsed in PBS and dissected under a Leica dissecting microscope using forceps to reveal the liver. For assessing BFP expression in *Tg(fabp10a:flox-pt-β-cat)* zebrafish, larvae were blocked for at least 1 h with PBS+4% BSA+0.3% Triton X-100 (PBT), and then incubated with chick anti-GFP primary antibody (1:500, Aves Cat #GFP-1020, Lot #0511FP12) for at least 12 h followed by AlexaFluor goat anti-chick 488 secondary antibody (A11039, Lot #1937504) for at least 12 additional hours. Larvae for all confocal imaging experiments were mounted in 1% low-melt agarose plus SlowFade Diamond Antifade Mountant (Invitrogen) and cover-slipped. All samples in each experiment were imaged on the same day on an Olympus confocal microscope using the same parameters for all zebrafish.

Quantification of BFP expression and Wnt reporter activity in 5-dpf *Tg(fabp10a:flox-pt-β-cat); Tg(7xTCF-Xla.Siam:nlsmCherry)* larvae was done on the same zebrafish. The second z-slice from the ventral surface of the liver (approximately 5 μm from the ventral surface) was selected for quantification. Image files were blinded and randomized. To quantify BFP expression (anti-GFP antibody recognizes BFP), the mean fluorescence in each liver was determined using FIJI/ImageJ (Rueden et al., 2017; Schindelin et al., 2012). To quantify Wnt reporter activity, blinded image files were scored manually for mCherry expression in hepatocytes according to the following scale: absent (no expression); low (expression in less than 10%); or high (expression in greater than 10%). This experiment was performed three times (three different clutches of zebrafish), with similar results each time. A representative example is shown for BFP expression, and pooled data is shown for Wnt reporter activity.

For assessing switching in *Tg(ubi:switch)* 6-dpf larvae, whole-mount imaging was performed twice (two different clutches of zebrafish), with similar results each time; representative images from one experiment are shown while data analysis was performed on the pooled data set of the two experiments.

### Assessing switching in 10-dpf, 20-dpf, and adult *Tg(fabp10a:CreERT2); Tg(ubi:switch)* zebrafish

Zebrafish at 10 or 20 dpf were fixed in 4% PFA for at least 12 h at 4°C, rinsed in PBS and then decalcified in 0.5 M pH 8 Ethylene Diamine Tetra Acetic Acid (EDTA) overnight at room temperature. Adult zebrafish (3–4 mpf) were euthanized 1 week post TAM treatment. Their livers and intestines were dissected from the body cavity and fixed in 4% PFA for at least 12 h at 4°C. Whole zebrafish larvae (10 or 20 dpf) and adult liver/intestines were immersed in 30% sucrose in PBS at 4°C, allowed to equilibrate, and cryosectioned into 5 μm-thick sections. On the day of imaging, frozen sections were air-dried for 30 min to 1 h; Slowfade Diamond Antifade Mountant was applied followed by cover-slipping and imaging on an Olympus IX81 Confocal microscope, using Olympus Fluoview version 4.1 software.

For quantification, the images were blinded using a randomization software and analyzed with ImageJ software. Hepatocytes were assigned and counted as either mCherry positive (switched) or EGFP positive (unswitched). Switching was assessed as the percentage of mCherry positive hepatocytes over total number of hepatocytes (mCherry positive plus EGFP positive). Genotyping for CreER was performed as described above using residual fish from the frozen block.

The 10-dpf experiment was performed three times (three different clutches of zebrafish), and data from three experiments were pooled. The 20-dpf experiment was performed once. The adult experiment was performed two times (two different clutches of zebrafish), and data from two experiments were pooled.

### Quantification of liver-to-body mass ratios and histological analysis

*Tg(fabp10a: CreER^T2^); Tg(fabp10a:flox-pt-β-cat), Tg(fabp10a: CreER^T2^)* and *Tg(fabp10a:flox-pt-β-cat)* zebrafish were treated with 10 µM TAM at 3 dpf or 2.3 µM TAM at 3 mpf and housed under standard conditions for 6 months post switching (until 6 mpf or 9 mpf, respectively). *Tg(fabp10a:pt-β-cat)* and non-transgenic control siblings were raised in parallel.

Fish were euthanized and weighed. Livers were dissected using a Leica dissecting microscope and weighed. These livers were fixed using 4% paraformaldehyde (PFA) in PBS for maximum 48 h at 4°C followed by immersion in 70% ethanol in embedding cassettes for paraffin embedding, sectioning (5 μm-thick sections) and H&E staining.

H&E-stained slides were blinded and scored by a board-certified pathologist (K.J.E.) based on established criteria for zebrafish HCC (Evason et al., 2015; Mudbhary et al., 2014). Samples were designated as follows: (1) HCC, defined by mild to severe cytologic abnormalities (nuclear contour irregularities, coarse chromatin, prominent nucleoli and/or increased nuclear-to-cytoplasmic ratios) and mild (irregular dilated sinusoids with intact bile ducts, focal compact growth/crowding), moderate (thickened cell plates, pseudoglands, large zones of compact growth without bile ducts) or severe (prominent pseudogland formation, zones with necrosis/apoptosis and very disrupted architecture) architectural abnormalities; (2) intermediate changes, defined by minimal/mild cytologic abnormalities and minimal/mild architectural abnormalities or moderate/severe cytologic abnormalities and no architectural abnormalities, and (3) no/minimal changes, defined by normal cytologic features and architecture or focal minimal/mild cytologic abnormalities or minimal/mild architectural abnormalities but not both. Oftentimes, multiple morphologically distinct lesions were observed within the same liver, sometimes with intervening liver parenchyma that appeared relatively normal. In these cases, only the highest-grade lesion was scored. Representative images of H&E-stained sections were taken using an Olympus BX53 microscope with attached Olympus DP73 camera and Olympus cellSens Entry 1.16 software.

This experiment was performed five times (five clutches of zebrafish) for *Tg(fabp10a: CreER^T2^); Tg(fabp10a:flox-pt-β-cat), Tg(fabp10a: CreER^T2^)* and *Tg(fabp10a:flox-pt-β-cat)* zebrafish. It was performed twice (two clutches of zebrafish) for *Tg(fabp10a:pt-β-cat)* and non-transgenic control siblings. After each experiment, H&E slides were blinded and scored in a preliminary round. After all experiments were complete, H&E slides from all experiments were blinded, randomized, and scored again. Any slides with a substantial difference in score (for example, HCC versus no/minimal changes) were blinded again and scored a third, definitive time.

### Measuring activated β-catenin and Wnt reporter activity in adult *Tg(fabp10a:pt-β-cat)* zebrafish

For quantifying Wnt reporter activity and β-catenin localization in adult zebrafish, progeny from a cross of *Tg(fabp10a:pt-β-cat)* and *Tg(7xTCF-Xla.Siam: mCherry)* zebrafish were raised to 6 mpf. The fish were weighed and their livers dissected for calculating liver-to-body mass ratios and cryosectioned for immunofluorescence imaging of antibody-stained β-catenin and reporter mCherry presence. Cryosections were air-dried for 30 min in the dark and washed in PBS, stained with mouse anti-β-catenin antibody (Sigma-Aldrich, Cat#C7207) (1:200 in 4% BSA-PBS) overnight at 4°C, washed five times with PBS, stained with AlexaFluor donkey anti-mouse 647 secondary (1:200 in 4% BSA-PBS, Invitrogen, Cat#A31571) at room temperature, and stained for 3 min with 5 µg/ml DAPI solution. Slides were then washed with PBS and mounted with SlowFade Diamond Antifade, cover-slipped, and imaged on an Olympus IX81 Confocal microscope and Olympus Fluoview version 4.1 software using a 405 nm laser for nuclear DAPI, 488 nm laser for β-catenin and 568 nm laser for the mCherry Wnt reporter. One field of view and z-slice was quantified per liver. Images in TIF format were analyzed using ImageJ after blinding image identity. Wnt reporter expression was scored manually as: (1) absent (no mCherry expression); (2) low (mCherry expression in less than 10% of cells); (3) high (mCherry expression in greater than 10% of cells). In terms of β-catenin localization, the samples were scored using the following matrix: 0, membrane only, wherein staining of membrane is distinct on all hepatocytes and much brighter than cytoplasm; 1+, focal (less than 10% of hepatic parenchyma) weak to moderate staining of hepatocyte cytoplasm with loss of distinct membrane staining; 2+, focal (less than 10% of hepatic parenchyma) strong cytoplasmic staining or patchy (10–50% of hepatic parenchyma) weak to moderate cytoplasmic staining; 3+, diffuse (more than 50% of hepatic parenchyma) staining of hepatocyte cytoplasm with loss of distinct membrane staining.

A subset of *Tg(fabp10a:pt-β-cat)* zebrafish and non-transgenic control siblings were only examined for β-catenin localization and not Wnt reporter activity. In these cases, immunofluorescence staining for β-catenin was performed on paraffin-embedded liver samples from one of the experiments that was used to assess HCC incidence in 6 mpf zebrafish. Paraffin-embedded sections were de-paraffinized by serial-immersion in three containers of Xylene for 5 min each, rehydrated by serial-immersion in two containers of 100% EtOH each, followed by 95% EtOH and 70% EtOH treatments, for 2 min each, ending with a 5 min ddH_2_O wash. Antigen retrieval was performed by treating the slides in a microwave oven (100% power setting) for 10 min in 10 mM citrate buffer pH 6.1. Before staining, sections were washed for 5 min in ddH_2_O. Pap pen was used to encircle the liver tissue fragment and blocking was done using PBT. Slides were stained for β-catenin using mouse anti-β-catenin antibody (1:200 in PBT) overnight at 4°C, washed four times with PBS+0.3% Triton X-100 (PT) for 15 min each, and stained with AlexaFluor donkey anti-mouse 647 secondary (1:200 in PBT, Invitrogen, Cat#A31571) overnight at 4°C. Slides were then washed four times with PT and allowed to dry completely for 30–60 min on benchtop. They were then mounted with SlowFade Diamond Antifade, cover-slipped, and imaged on an Olympus IX81 Confocal microscope and Olympus Fluoview version 4.1 software using a 647 nm laser for β-catenin. Two to five fields of view were taken per fish and all of them were analyzed using one z-slice each per field of view. Images in TIF format were blinded, analyzed and scored for β-catenin localization using the matrix described above. In cases where the diagnosis was different for multiple field of views from the same fish, the majority diagnosis was taken as final diagnosis for that sample.

### RNA seq

Zebrafish of the following genotypes/treatment groups were used as tissue source: *Tg(fabp10a:CreERT2); Tg(fabp10a:flox-pt-β-cat)* zebrafish treated with TAM from 3–6 dpf (CreLox), with HCC (*n*=3); *Tg(fabp10a:CreERT2); Tg(fabp10a:flox-pt-β-cat)* zebrafish treated with TAM from 3–6 dpf (CreLox), without HCC (*n*=2); and *Tg(fabp10a:CreERT2)* zebrafish treated with ethanol from 3–6 dpf (sibling controls), without HCC (*n*=4). All these fish were male, and were euthanized at 11 mpf by rapid chilling. Their livers were dissected for assessment of liver-to-body mass ratios, and half of each liver was submitted for histologic analysis as described above. The other half of each dissected liver was flash-frozen on dry ice and stored in −80°C prior to RNA extraction. RNA was isolated using TriReagent (Cat# R2050-1-50, Zymo Research) and Direct-Zol RNA Mini prep kit (Cat# R2050, Zymo Research) including DNase treatment following the manufacturer's protocols. Samples were eluted in RNase-/DNase-free water. Sample quality control, library preparation, sequencing and alignments were performed by the Huntsman Cancer Institute (HCI) High Throughput Genomics and Bioinformatic Analysis Shared Resource. Paired-read, 150 base pair sequencing was performed using Illumina's NovaSeq instrument. Three or four pooled libraries were run on each lane of the flow cell.

We performed a similar RNA sequencing experiment using livers isolated and prepared as described above from 6-mpf *Tg(fabp10a:pt-β-cat)* (HepABC) zebrafish with HCC (*n*=5) and their non-transgenic siblings without HCC (*n*=5). Three to four pooled libraries were run on each lane of the flow cell.

### Single-cell RNA-sequencing using the 10× Chromium platform

The following 6-mpf male zebrafish were used for this experiment: *Tg(fabp10a*:*pt-β-cat); Tg(7xTCF-Xla.Siam: mCherry)* [HCC(HepABC)], *Tg(fabp10a:CreERT2; fabp10a:flox-pt-β-cat)* zebrafish treated with TAM from 3–6 dpf [HCC(CreLox)], and *Tg(fabp10a:flox-pt-β-cat)* treated with TAM from 3-6 dpf (no HCC). Zebrafish were euthanized by rapid chilling and their livers were dissected. Half of each liver was submitted for histologic evaluation to confirm the diagnosis (HCC or no HCC). The remaining half of each liver was dissociated into single-cell suspensions and prepared for single-tube single-cell RNA sequencing (scRNA-seq) based on the 10× Genomics platform. Dissected liver tissue was immersed in 5% Fetal Bovine Serum (FBS) in Hank's Buffered Saline Solution (HBSS) without Phenol Red, with calcium and magnesium and chopped finely. The cells were homogenized in 1 ml of 0.25% trypsin+EDTA for 5 min at room temperature and re-suspended in 1 ml of 5%FBS+5 mM EDTA in HBSS-Free solution. Cells were then filtered through a 40 μm membrane filter, and the filtrate was centrifuged at 1200 RPM, 4°C for 5 min. The cell pellet was then re-suspended in phosphate buffered saline with 0.04% bovine serum albumin. The cell suspension was then filtered again through 40 μm cell strainers to obtain a liver single-cell suspension.

Samples were submitted to the HCI High-Throughput Genomics Shared Resource, where viability and cell count were assessed on Countess I (Thermo Fisher Scientific). The Chromium Single Cell Gene Expression Solution with 3′ chemistry, version 2 (PN-120237) was used to barcode individual cells with 16 bp 10× Barcode and to tag cell specific transcript molecules with 10 bp Unique Molecular Identifier (UMI) according to the manufacturer’s instructions. Equilibrium to targeted cell recovery of 6000 cells along with 10× Gel Beads and reverse transcription reagents were loaded to Chromium Single Cell A Chip (PN-120236) to form Gel-Bead-In EMulsions (GEMs), the micro-droplets. Within individual GEMs, cDNA generated from captured and barcoded mRNA was synthesized by reverse transcription at the setting of 53°C for 45 min followed by 85°C for 5 min. Subsequent A tailing, end repair, adaptor ligation and sample indexing was performed in bulk according to the manufacturer's instructions. The resulting barcoding libraries were qualified on Agilent D1000 ScreenTape on Agilent Technology 2200 TapeStation system and quantified by quantification PCR using KAPA Biosystems Library Quantification Kit for Illumine Platforms (KK4842). Multiple libraries were then normalized and sequenced on NovaSeq 6000 with 2×150 PE mode.

All protocols to generate scRNA-seq data on 10× Chromium platform including library prep, instrument and sequencing setting can be found on: https://support.10xgenomics.com/single-cell-gene-expression.

### Bioinformatics

#### Bulk RNA sequencing

For the CreLox and corresponding control zebrafish, optical duplicates were filtered out with BBMap's clumpify utility (v38.34). Sequence adapters were removed with cutadapt (v1.16). Raw FASTQ data was aligned with STAR (v2.6.1b) to the zebrafish genome (build GRCz11) with splice junctions annotated from Ensembl release 94. Gene expression was quantified with featureCounts (v1.6.3). Differential gene expression analysis was performed with DESeq2 (v1.22.2). Pipeline specifics and code are documented on GitHub (https://github.com/smkalasekar/Heterogeneous-beta-catenin-activation-is-sufficient-to-cause-hepatocellular-carcinoma-in-zebrafish).

For the HepABC and corresponding control zebrafish, sequence adapters were removed with cutadapt (v1.16). Raw FASTQ data was aligned with STAR (v2.6.1b) to the zebrafish genome (build GRCz11) with splice junctions annotated from Ensembl release 94. Gene expression was quantified with featureCounts (v1.6.3). Differential gene expression analysis was performed with DESeq2 (v1.20.0). Pipeline specifics and code are documented on GitHub (https://github.com/smkalasekar/Heterogeneous-beta-catenin-activation-is-sufficient-to-cause-hepatocellular-carcinoma-in-zebrafish).

Data from the following differential gene expression analyses were used for subsequent investigation including pathway analysis and generation of Venn diagrams: (1) CreLox(HCC)=CreLox with HCC versus control siblings (Cre driver only) without HCC; (2) NoHCC=CreLox without HCC versus control siblings (Cre driver only) without HCC; and (3) HepABC(HCC)=HepABC with HCC versus non-transgenic siblings without HCC.

Data from the aforementioned comparisons were analyzed through the use of Ingenuity Pathway Analysis (IPA), using the log ratios of molecules with significantly (*P*<0.05) altered gene expression as inputs. Canonical Pathways Analysis was used to identify the pathways from the IPA library of Canonical Pathways that were most significant to the data set. Functional Analysis was used to identify the biological functions and/or diseases that were most significant to the data set and were associated with biological functions and/or diseases in Ingenuity's Knowledge Base. IPA calculates *P*-values using Fisher's exact test. For Table S2, all three datasets were fed into the Comparison Analysis feature of IPA. Heatmaps from the comparison analyses were used to identify pathways commonly dysregulated in three samples. The *P*-values were derived from each individual dataset's Summary or Functional Analysis outputs generated by the IPA software.

Proportional Venn diagrams in Fig. 6 were plotted using an R Shiny app (http://eulerr.co/). *P*-values for the Venn overlap significance were calculated using the *dhyper* tool in R (https://rdrr.io/bioc/GeneOverlap/man/GeneOverlap.html). For calculations, the total number of genes in the genome was considered as 25,903 (Ensembl Zebrafish GRCz10).

#### Single-cell RNA sequencing

10× Genomics' Cell Ranger software (v2.2.0) executed primary data analysis through a series of bioinformatics pipelines using a combination of custom and third-party tools. Pipeline ‘cellranger mkfastq’ converted raw sequencing data into de-multiplexed FASTQ files with Illumina's bcl2fastq software. Two custom transgenic genomic references were built with ‘cellranger mkref’, but shared the common *Danio rerio* genome reference build GRCz11 with annotations from Ensembl release 94. The Ensembl gene annotations were filtered with ‘cellranger mkgtf’ for gene_biotypes matching ‘protein_coding’, ‘lincRNA’ and ‘antisense’ tags. All samples had additional transgenic sequence/annotations added: Xenopus β-catenin and EBFP sequences were added for HCC(CreLox) (X2) and no HCC(X4) samples, and Xenopus β-catenin sequence was added for the HCC(HepABC) sample (X1).

Each sample was processed with ‘cellranger count’ pipeline with their respective transgenic genome build with parameter ‘--expect-cells=3000’. Quality control (QC) reports from 10x Genomics reported warnings regarding the low fraction of reads in cells because its cell versus background algorithm assumes the cells' captured RNA content is within a single order of magnitude. However, the total UMI count versus ranked barcode plots (not shown) exhibited a more gradual sloping behavior than expected, which means the default Cell Ranger algorithm (v2.2.0) classifying cells versus background GEMs left out potential cell-like GEM partitions with much less RNA content due to either biological or technical reasons. In an attempt to recover those (perhaps lower quality) GEM partitions, the raw gene-barcode matrices from ‘cellranger count’ (located in ‘outs/raw_gene_bc_matrices’) were processed with the EmptyDrops algorithm (R package DropletUtils v1.2.2) to discriminate cells from background GEM partitions at a false discovery rate (FDR) of 1% (Lun et al., 2019). GEM partitions with 500 UMI counts or less were considered to be devoid of viable cells, while those with at least 10,000 UMI counts were automatically considered to be cells. For each sample, the ambient RNA's *P*-value null distribution was confirmed to be approximately uniform.

Cell-based QC metrics were calculated with R package scater (v1.10.1) using the calculateQCMetrics function (McCarthy et al., 2017). Principal component analysis (PCA) on the cell-based QC metrics combined with a multivariate outlier method flagged cells with outlying values in QC metrics as suspect (Filzmoser et al., 2008). Cells with extremely low UMI counts, extremely low gene counts, or extremely high percentage of expression attributed to mitochondrial genes were also flagged as low quality. Extremeness in any of these three measures was determined by three median absolute deviations from the median with the scater::isOutlier function applied to each sample individually. These cells suspected of being low quality were removed from downstream analysis. Characteristics of cells leading to their elimination are enumerated in Table S18. No cells exhibited extreme low UMI or gene count, while some sickly cells were dominated by extremely high mitochondrial expression. Many putative cells were removed because PCA analysis of several QC metrics indicated unusual characteristics. However, more rigorous filters based with more stringent UMI and gene count cutoffs were applied via custom R algorithms (Github, https://github.com/smkalasekar/Heterogeneous-beta-catenin-activation-is-sufficient-to-cause-hepatocellular-carcinoma-in-zebrafish). While the PCA-based outlier filter is quite aggressive, the number of cells passing QC still far exceeds the default from 10× Genomics naïve classification of cells.

Seurat V3 software (Butler et al., 2018; Stuart et al., 2018preprint) was used to analyze all data. Customized algorithms, which are all based on Seurat workflows, are described in detail at Github (https://github.com/smkalasekar/Heterogeneous-beta-catenin-activation-is-sufficient-to-cause-hepatocellular-carcinoma-in-zebrafish). Single-cell gene expression data is also publicly accessible on the Gene Expression Omnibus (GSE137788).

Following QC filters and data transformation to regress out variations driven by cells with high mitochondrial transcriptional activity, transcripts from a total of 1077 cells from no HCC control, 2112 cells from HCC(CreLox), and 2866 cells from HCC(HepABC) were used for subsequent analysis. After gene expression normalization, we performed PCA on the 2000 most highly variable genes in each sample as recommended by the Seurat V3 pipeline (Stuart et al., 2018preprint). Principal components (PCs) were then analyzed, and PCs up to 12 for HCC(HepABC), 16 for HCC(CreLox), and 16 for no HCC control were identified as relevant for subsequent clustering analyses based on statistical significance and minimal standard deviation from JackStraw and Elbow plots.

The graph-based t-Distributed Stochastic Neighbor Embedding (t-SNE) approach was executed for linear dimensional reduction of the chosen principal components (PCs) (Mwangi et al., 2014; van der Maaten, 2014). In the resultant t-Distributed Stochastic Neighbor Embedding (t-SNE) plots, spatial relationships between any two points/color-coded clusters are indicative of the transcriptional distances between them. Seurat algorithms were further applied to compute the most highly enriched genes per cluster in order to determine cellular identity. Cluster cell types were estimated based on computed lists of enriched genes as well as significantly differentially expressed genes previously known to be upregulated in each cell type (Spanjaard et al., 2018). Custom R scripts (available on Github, https://github.com/smkalasekar/Heterogeneous-beta-catenin-activation-is-sufficient-to-cause-hepatocellular-carcinoma-in-zebrafish) were used to identify the markers used to predict cluster composition of t-SNE plots derived from No HCC, HCC(CreLox), HCC(HepABC) zebrafish, and all three samples combined (Tables S19–S22), as well to identify upregulated genes within specific clusters in these plots (Tables S10, S12, S14).

The DAVID Genome Browser (Huang et al., 2009a,b) was used to perform Gene Ontology analysis of the identified cluster-specific markers (Tables S13, S15, S17). Custom R scripts were also used to compute the percentage cluster-wise representation of cells from each sample (Table S11), percentage of cells from each sample expressing 0, 1, 2, 3 or 4 Wnt targets *axin2*, *mtor*, *glula*, *myca* and *wif1* (Table S16) and *jun* expression in cells isolated from livers of Non-HCC, CreLox HCC, and HepABC HCC (Table S17).

### General methodology, image analysis and statistical methods

For each experiment involving tumor formation in adult zebrafish, an entire clutch of zebrafish was raised, so sample size was dependent on the size of the clutch. Animals that died before the experimental endpoint were excluded from analysis; this exclusion criteria was pre-established. All other animals were included in analysis. For each larval imaging experiment, we typically included five to ten zebrafish per genotype/group, based on the presence of fluorescent marker(s). Zebrafish with livers that were disrupted or lost during processing were excluded from analysis; this exclusion criteria was pre-established.

Immunofluorescence imaging was performed using an Olympus IX81 Confocal microscope with Olympus Fluoview version 4.1 software. Within each experiment, the same parameters (HV, gain, offset, etc.) were maintained for all images. Images were blinded and randomized using R (R Core Team, 2017), and then analyzed with FIJI/ImageJ (Rueden et al., 2017; Schindelin et al., 2012). For representative images, any adjustments in contrast were performed in parallel to all images within a figure panel using ImageJ to maintain the same parameters across images.

Data were analyzed and graphs were generated using GraphPad Prism software (version 7.04 or 8.0). To compare the extent of Cre-mediated switching among different genotypic and treatment groups and liver-to-body mass ratios an ordinary one-way ANOVA with Sidak's multiple comparisons test was performed. Alternatively, when standard deviations of groups were significantly different, non-parametric Kruskal–Wallis test followed by Dunn's multiple comparisons test was performed. To compare cytoplasmic β-catenin localization, Wnt reporter activity, and incidence of HCC and intermediate changes, Fisher's exact test was performed. Differences were considered statistically significant if *P*-values were less than 0.05. Mean values with standard deviations are reported for each experiment, unless otherwise mentioned.

## Supplementary Material

Supplementary information
