# Supplementary material for: Heterogeneous beta-catenin activation is sufficient to cause hepatocellular carcinoma in zebrafish
Source: Biol Open. 2019 Oct 1;8(10):bio047829. doi: 10.1242/bio.047829 (PMC6826293; doi:10.1242/bio.047829)
Supplement: Supplementary information [file biolopen-8-047829-s1.pdf]

Fig. S1

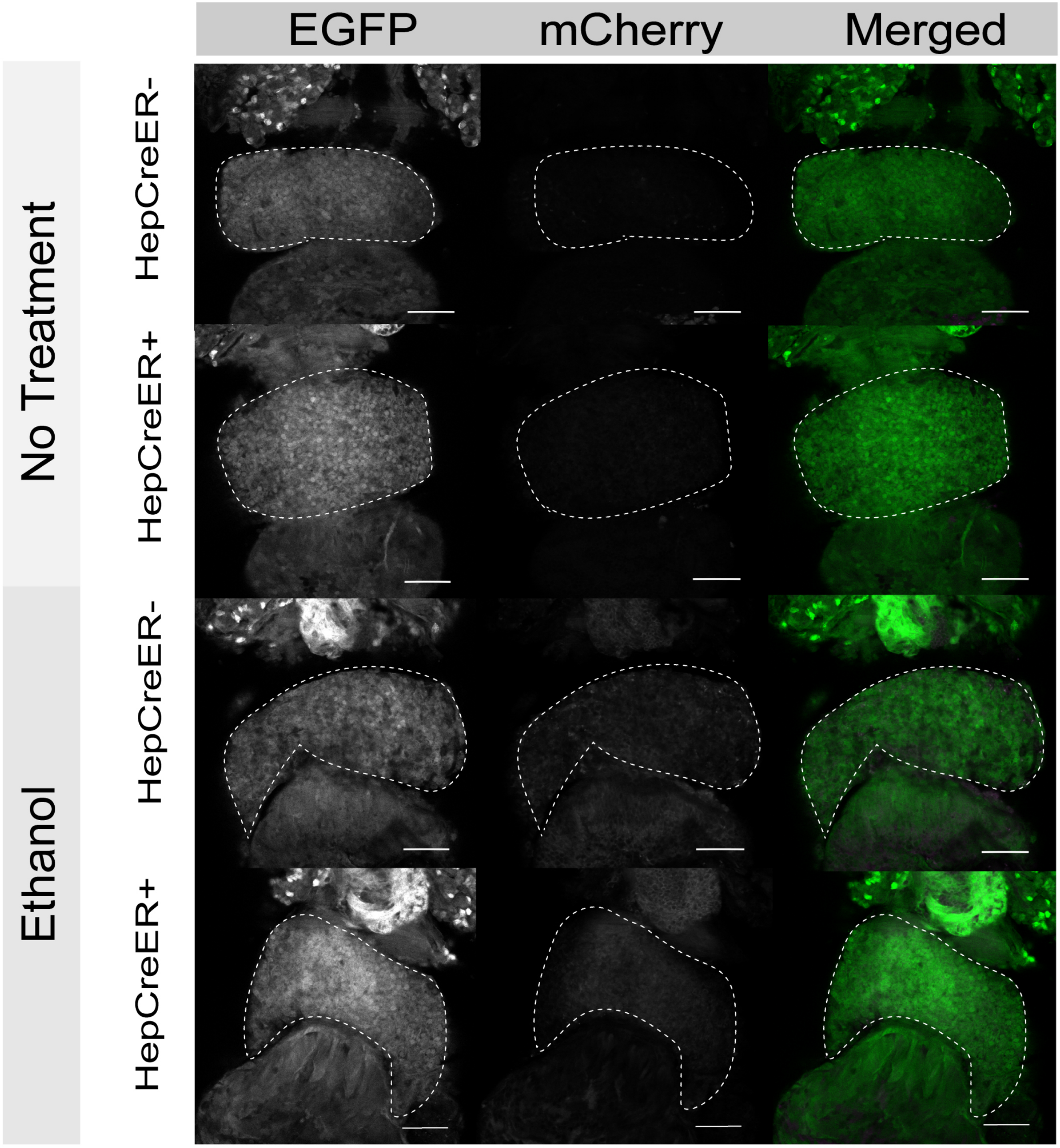

Figure S1: Switching in CreERT2 modulated switch system at 6 dpf. Representative images of *Tg(ubi:switch)* (HepCreER-) and *Tg(fabp10a:CreERT2); Tg(ubi:switch)* (HepCreER+) larvae treated with ethanol or egg water only (no treatment) between 3 dpf to 6 dpf and imaged at 6 dpf for EGFP and mCherry expression. No hepatocytes in these images have switched; all have EGFP expression and lack mCherry expression. Livers have been outlined with white dotted lines. Scale bars indicate 50 µm. This experiment was performed twice and representative images are from one experiment.

Fig. S2

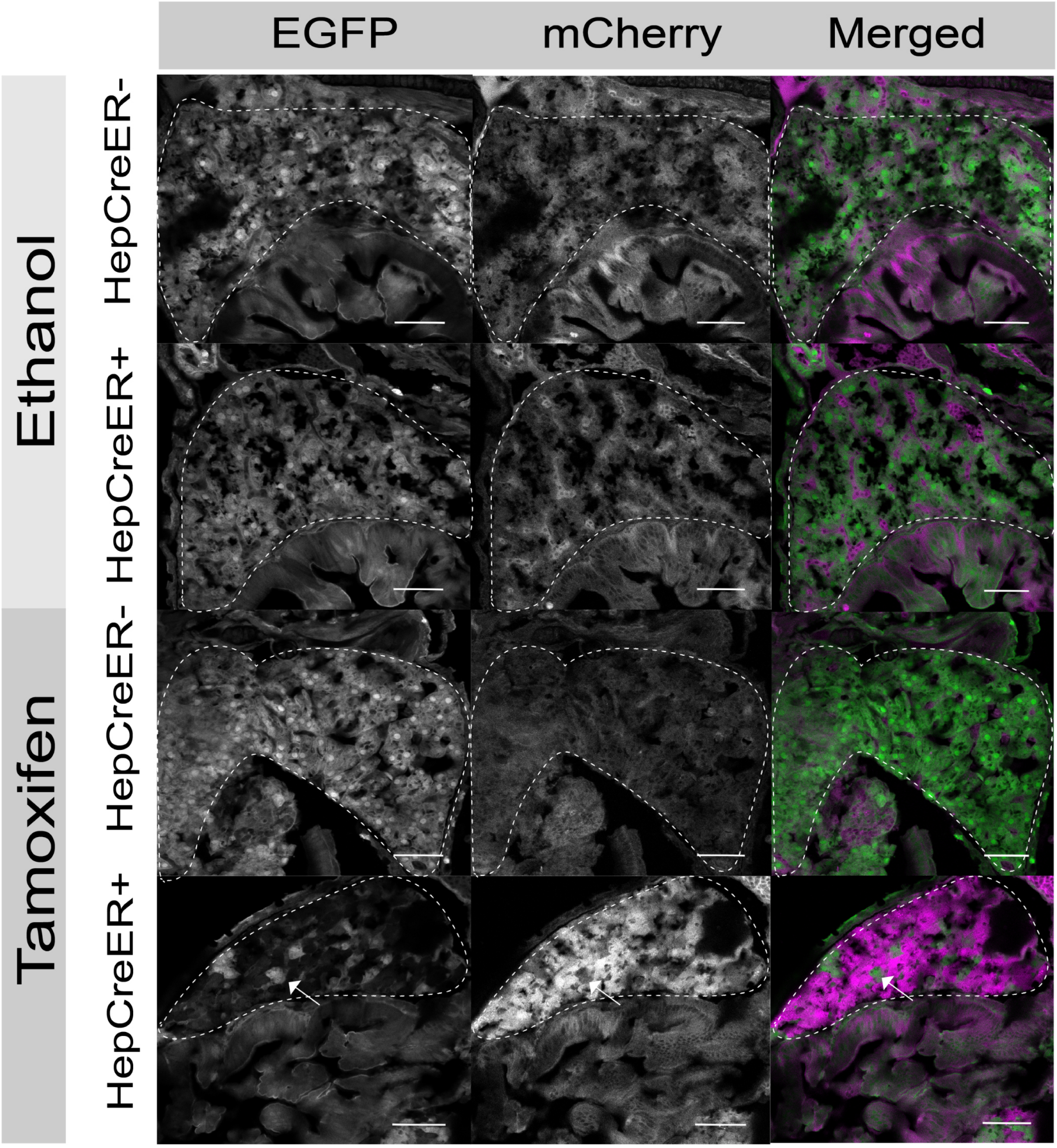

Figure S2: Switching in CreERT2 modulated switch system at 10 dpf. Representative images of *Tg(ubi:switch)* (HepCreER-) and *Tg(fabp10a:CreERT2); Tg(ubi:switch)* (HepCreER+) larvae treated with 4-hydroxytamoxifen (tamoxifen) or ethanol between 3 dpf to 6 dpf and imaged at 10 dpf for EGFP and mCherry expression. Almost all hepatocytes have switched in the Tamoxifen-treated HepCreER+ larva (have mCherry expression and lack EGFP expression) and unswitched hepatocytes are indicated by white arrows. Livers have been outlined with white dotted lines. Scale bars indicate 50 μm. This experiment was performed thrice and representative images are from one experiment.

Biology Open • Supplementary information

Fig. S3

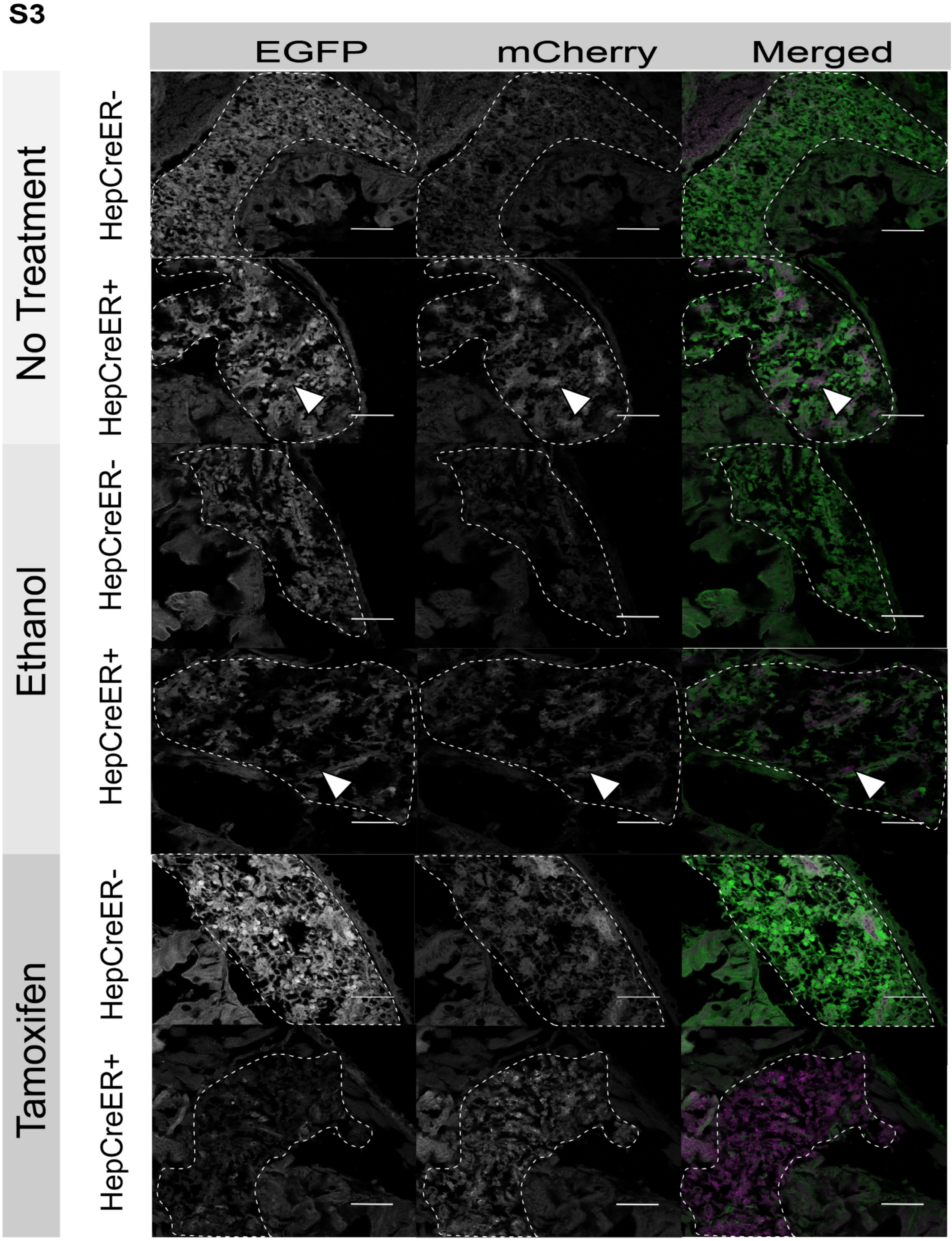

Figure S3: Switching in CreERT2 modulated switch system at 20 dpf. Representative images of *Tg(ubi:switch)* (HepCreER-) and *Tg(fabp10a:CreERT2); Tg(ubi:switch)* (HepCreER+) larvae treated with 4-hydroxytamoxifen (tamoxifen), ethanol, or egg water alone (no treatment) between 3 dpf to 6 dpf and imaged at 20 dpf for EGFP and mCherry expression. Successful switching is indicated by the loss of EGFP expression and gain of mCherry expression (arrowheads). All hepatocytes in tamoxifen-treated HepCreER+ liver have switched (bottom panels). Livers have been outlined with white dotted lines. Scale bars indicate 50µm. This experiment was performed once.

Biology Open (2019): doi:10.1242/bio.047829: Supplementary information

Fig. S4

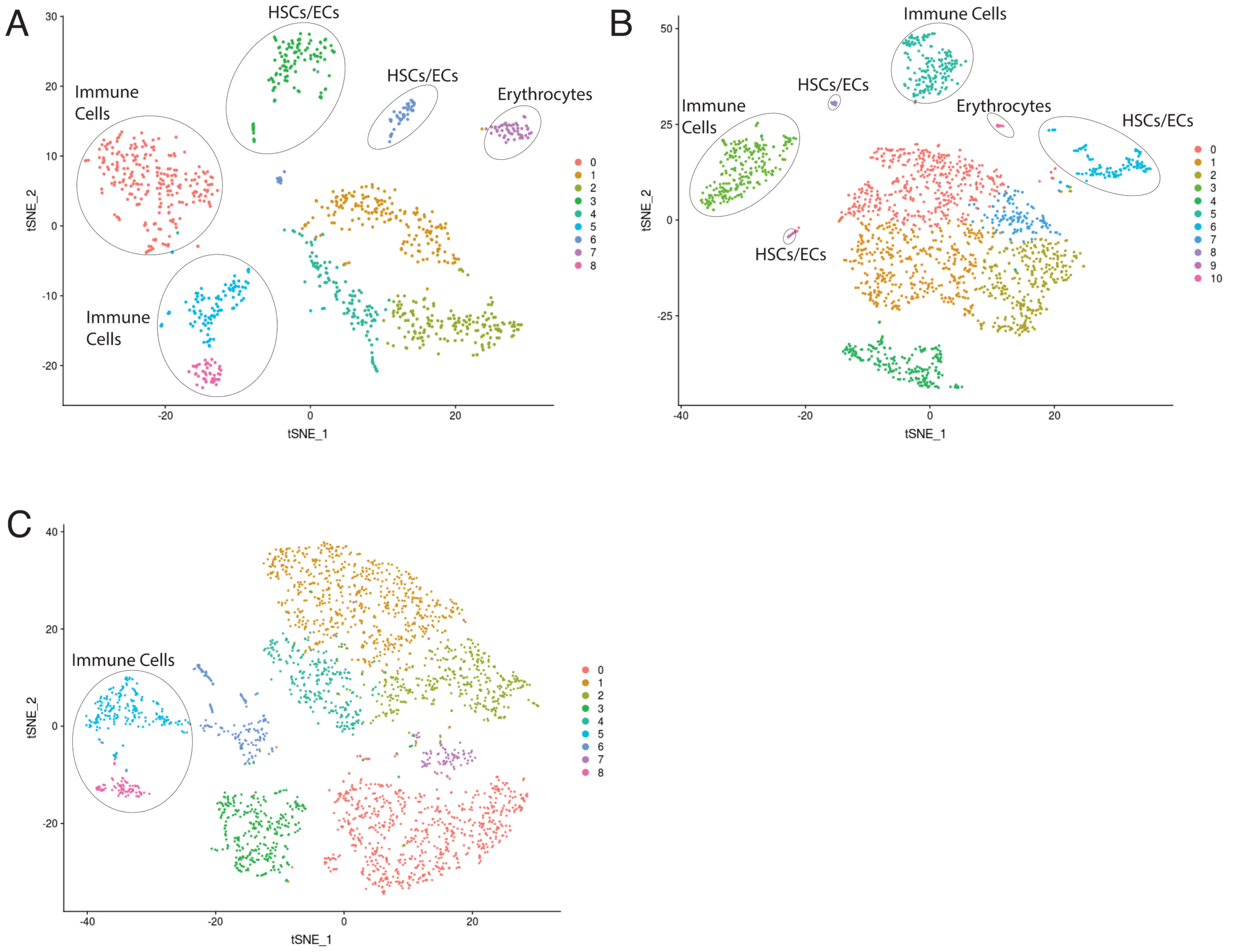

Figure S4: Single-cell RNA sequencing of zebrafish liver and β-catenin-driven HCC. (**A**) t-SNE plot of 1077 cells isolated from the liver of 6-mpf male zebrafish without HCC. Cells are color-coded by their associated cluster. Non-hepatocytes are indicated as identified by the expression of markers highlighted in Table S18. All non-circled cells are hepatocytes. (**B**) t-SNE plot of 2112 cells isolated from the liver of 6-mpf male CreLox zebrafish with HCC. Cells are color-coded by their associated cluster. Non-hepatocytes are indicated as identified by the expression of markers highlighted in Table S19. All non-circled cells are hepatocytes. (**C**) t-SNE plot of 2866 cells isolated from the liver of 6-mpf male *Tg(fabp10a:pt-B-cat)* (HepABC) zebrafish with HCC. Cells are color-coded by their associated cluster. Non-hepatocytes are indicated as identified by the expression of markers highlighted in Table S20. All non-circled cells are hepatocytes.

Biology Open • Supplementary information

Supplementary Tables

Table S1: Transgenic lines

Full name of line	Abbreviation (text)	Abbreviation (Figures)	Purpose	Reference
Tg(fabp10a:Xla.Ctnnb1, cryaa:Venus)	*Tg(fabp10a:pt-β-cat)*	HepABC	Established activated β-catenin-driven HCC model	Evason et al., 2015
Tg(7xTCF-Xla.Siam:nlsmCherry)	*Tg(7xTCF-Xla.Siam:nlsmCherry)*	Wnt Reporter	Reporter for activated β-catenin-driven transcription	Moro et al., 2012
Tg(-3.5ubb: LOXP-EGFP-LOXP-mCherry)	*Tg(ubi:loxP-EGFP-loxP-mCherry)* or *Tg(ubi:switch)*		Reporter line to characterize TAM-induced recombination	Mosimann et al., 2011
Tg(fabp10a:Cre, cryaa:Venus)	*Tg(fabp10a:Cre)*	HepCre	To test functional recombination of *Tg(fabp10a:flox-pt-β-cat)* lox switch.	Ni et al., 2012
Tg(fabp10a:Cre-ERT2,cryaa:Venus)	*Tg(fabp10a:Cre-ERT2)*	HepCreER	Temporal control of hepatocyte-specific Cre recombination.	
Tg(fabp10a:LOXP-BFP-LOXP-Xla.Ctnnb1, cryaa:mCherry)	*Tg(fabp10a: flox-pt-β-cat)*	FloxABC	Lox switch enabling activated β-catenin activation upon Cre-induced recombination.	
Tg(fabp10a:Cre-ERT2,cryaa:Venus); Tg(fabp10a:LOXP-BFP-LOXP-Xla.Ctnnb1, cryaa:mCherry)	CreLox	CreLox	Test progeny in which TAM-induction should cause Cre recombination and oncogene expression.	

Biology Open • Supplementary information

Table S2: Ingenuity pathway analysis of significantly differentially expressed genes in CreLox zebrafish with HCC, HepABC zebrafish with HCC, and CreLox zebrafish without HCC.

Name	Category	p-value in individual dataset		
		CreLox HCC	HepABC HCC	CreLox no HCC
Wnt/B-catenin signaling pathway	Canonical Pathways	6.34E-03	8.34E-07	N.S.
Liver Tumor	Diseases and Functions	2.87E-11	9.77E-19	N.S.
Inflammation of Liver	Diseases and Functions	5.22E-04	2.74E-04	N.S.
Cancer	Diseases and Disorders	2.80E-05 – 5.19E-37	7.91E-09 - 4.38E-133	3.54E-03 - 2.17E-10
Organismal Survival	Physiological System Development and Function	2.05E-10 - 5.65E-11	4.63E-09 - 6.58E-46	5.11E-06
Liver hyperplasia/hyperproliferation	Tox Functions	1.00E00 - 3.16E-14	4.51E-01 - 2.67E-13	5.43E-01 - 3.81E-05
Liver Steatosis	Tox Functions	1.78E-01 - 2.68E-09	1.00E00 - 1.34E-08	2.71E-01 - 7.88E-04

N.S. Not significant.

Biology Open • Supplementary information

Table S3. Transcripts significantly dysregulated in CreLox zebrafish with HCC but not in CreLox zebrafish without HCC.

Click here to Download Table S3

Table S4. Transcripts significantly dysregulated in CreLox zebrafish with HCC and in CreLox zebrafish without HCC.

Click here to Download Table S4

Table S5. Transcripts significantly dysregulated in CreLox zebrafish without HCC but not in CreLox zebrafish with HCC.

Click here to Download Table S5

Table S6: Transcripts significantly dysregulated in CreLox zebrafish with HCC and in HepABC zebrafish with HCC.

Click here to Download Table S6

Table S7: Transcripts significantly dysregulated in CreLox zebrafish with HCC but not in HepABC zebrafish with HCC.

Click here to Download Table S7

Table S8: Transcripts significantly dysregulated in HepABC zebrafish with HCC but not in CreLox zebrafish with HCC.

Click here to Download Table S8

Biology Open • Supplementary information

Table S9: Percentage cluster-wise representation of cells from Non-HCC, CreLox HCC, and HepABC HCC in integrated t-SNE plot combining all samples.

Cluster in Integrated Analysis	Non-HCC %	CreLox %	HepABC %	Total
0	18.06	75.52	6.42	100
1	4.92	13.52	81.57	100
2	41.13	43.39	15.48	100
3	13.95	8.16	77.89	100
4	8.71	8.33	82.95	100
5	6.29	83.57	10.14	100
6	5.8	22.54	71.65	100
7	5.76	0.92	93.32	100
8	13.51	23.51	62.97	100
9	30.32	43.44	26.24	100
10	39.56	44.55	15.89	100
11	22.12	21.63	56.25	100
12	53.62	21.74	24.64	100
13	79.37	20.63	0	100

Biology Open • Supplementary information

Table S10: Genes up-regulated in Cluster 5 of the t-SNE plot consisting of livers from HCC (HepABC), HCC (CreLox) and No HCC zebrafish.

afp4.1
agxtb
ahcy
aldob
anxa4
apoa1a
apoa4b.1
basp1
bhmt
BX908782.2
cdab
cebpa
chia.6.1
ckba
diabloa
eps8l3a
fabp3
fabp7a
fads2
fdps
fhl1b
ftcd
g6pca.1
gamt
gpx4a
gstt1a
hmgcra
hmgcs1
hpda
icn
krt18
krt8
ldhbb
lgals2b
me1
mibp2
mid1ip1b
miox
msmo1
mt2
nme4
pck1
pklr
pla2g12b
rgs4
rgs5a
rpl22l1
s100a10a
s100a10b
s100v2
serpina7
serpinb1
si:dkey-7f3.14
si:dkey-86l18.10
si:dkeyp-73d8.6
si:dkeyp-73d8.9
tdo2a
tmem97
tpt1
tubb2b
txn
uchl1
uox
uraha
zgc:77439
zgc:85843

Biology Open • Supplementary information

Table S11. Gene Ontology analysis of genes up-regulated in Cluster 5 of the t-SNE plot consisting of livers from HCC (HepABC), HCC (CreLox) and No HCC zebrafish.

Click here to Download Table S11

Table S12: Genes up-regulated in Cluster 4 of the t-SNE plot consisting of livers from HCC (HepABC), HCC (CreLox) and No HCC zebrafish.

afp4.1	apoc2	gpx4a	si:ch73-281k2.5
agt	apom	hp.1	si:dkey-90m5.4
ahcy	cbln8	hpx	si:dkeyp-73d8.6
ahsg1	ces2	kng1	si:dkeyp-73d8.9
ahsg2	cfhl4	leg1.1	tfa
aldob	cfhl5	pla2g12b	wu:fj16a03
ambp	cyp2ad2	serpina1	zgc:123103
apoa1a	f2	serpina11	zgc:174259
apoa1b	fetub	serpina7	zgc:66313
apoa2	fga	serpinc1	zgc:77439
apoa4b.1	fgb	serping1	
apoc1	fgg	si:ch211-186e20.7	

Table S13. Gene Ontology analysis of genes up-regulated in Cluster 4 of the t-SNE plot consisting of livers from HCC (HepABC), HCC (CreLox) and No HCC zebrafish.

Term	Count	PValue	Genes	Fold Enrichment	Bonferroni	Benjamini	FDR
GO:0004867~serine-type endopeptidase inhibitor activity	9	0.00	AMBP, SERPINA7, SI:CH211-186E20.7, AGT, SERPINC1, SERPINA1, SERPING1, SERPINA1L, ZGC:174259	56.69	0.00	0.00	0.00
GO:0004869~cysteine-type endopeptidase inhibitor activity	4	0.00	KNG1, FETUB, AHSG1, AHSG2	112.63	0.00	0.00	0.01
GO:0030674~protein binding, bridging	3	0.00	FGG, FGA, FGB	130.55	0.02	0.01	0.22
GO:0004866~endopeptidase inhibitor activity	3	0.00	FETUB, AHSG1, AHSG2	43.52	0.13	0.04	2.08
GO:0031210~phosphatidylcholine binding	2	0.02	APOA1B, APOA1A	119.67	0.68	0.21	15.50
GO:0060228~phosphatidylcholine-sterol O-acyltransferase activator activity	2	0.02	APOA1B, APOA1A	106.38	0.73	0.19	17.26
GO:0008289~lipid binding	3	0.02	APOA4B.1, APOA1B, APOA1A	13.42	0.76	0.18	18.70
GO:0015485~cholesterol binding	2	0.03	APOA1B, APOA1A	59.84	0.90	0.25	28.60
GO:0017127~cholesterol transporter activity	2	0.03	APOA1B, APOA1A	59.84	0.90	0.25	28.60
GO:0005102~receptor binding	3	0.07	FGG, FGA, FGB	6.50	1.00	0.45	54.71

Biology Open • Supplementary information

Table S14: Genes up-regulated in Cluster 7 of the t-SNE plot consisting of livers from HCC (HepABC), HCC (CreLox) and No HCC zebrafish.

a2ml	c3a.6	fetub	serpina1
ahsg1	c9	fga	serpina1l
ahsg2	cbln8	fgb	si:ch211-212c13.8
ambp	ces2	fgg	si:ch73-281k2.5
apoa1b	ces3	fn1b	si:dkeyp-73d8.6
apoa2	cfb	hpx	si:dkeyp-73d8.9
apoa4b.1	cfhl4	itih3a	slc38a4
apoba	cp	kng1	tfa
apobb.1	crp2	leg1.1	ugt1a2
apom	crp3	p4hb	wu:fj16a03
bfb	cyp1a	pck2	zgc:112265
c3a.1	cyp2ad2	plg	zgc:123103
c3a.2	f2	rltgr	zgc:153921.1
c3a.3	f5	rrbp1b	

Table S15. Gene Ontology analysis of genes up-regulated in Cluster 7 of the t-SNE plot consisting of livers from HCC (HepABC), HCC (CreLox) and No HCC zebrafish.

Click here to Download Table S15

Biology Open • Supplementary information

Table S16: Percentage of cells from each sample expressing 0, 1, 2, 3 or 4 Wnt targets *axin2, mtor, glula, myca, and wif1*.

**Number of Wnt targets**	**No HCC**	**CreLox HCC**	**HepABC HCC**
0	89.6	86.8	44.3
1	9.3	10.3	40.5
2	1.1	2.1	13
3	0	0.8	2
4	0	0	0.2
**Total %**	100	100	100

Biology Open • Supplementary information

Table S17*: jun* expression in cells isolated from livers of Non-HCC, CreLox HCC, and HepABC HCC

	% cells expressing *jun*	Average expression level (arbitrary log normalized unit *)	*p* value (vs. no HCC)
No HCC	52.6	3.717897	
HCC (CreLox)	21	4.799917	< 0.0001
HCC (HepABC)	83.54	3.81732	0.0004

* Units were log normalized using Seurat's Log10 normalization method.

Table S18: Cell quality control filtering statistics

Sample	SampleID	Cells Passing QC Filters	Low UMI count	Low gene count	High % Mitochondrial	PCA of QC metrics outliers
HCC (HepABC)	15547X1	5830	0	0	1380	1720
HCC (CreLox)	15547X2	2785	0	0	1307	1253
No HCC	15547X4	3218	0	0	11	230

Table S19: Markers used to predict cluster composition in livers from No HCC zebrafish.

Click here to Download Table S19

Table S20: Markers used to predict cluster composition in livers from HCC (CreLox) zebrafish.

Click here to Download Table S20

Biology Open • Supplementary information

Table S21: Markers used to predict cluster composition in livers from HCC (HepABC) zebrafish.

Gene	Log fold change (average)	P value (adjusted)	Cluster	Predicted Cell Type
ahsg1	0.30684514	1.50E-50	0	Hepatocytes
hpx	0.39879044	1.81E-45	0	Hepatocytes
afp4.1	0.74106153	3.10E-228	1	Hepatocytes
ahsg1	0.28787103	4.46E-21	2	Hepatocytes
apobb.1	0.26714745	1.07E-15	2	Hepatocytes
fetub	0.31855413	1.19E-25	2	Hepatocytes
hpx	0.55067028	9.91E-47	2	Hepatocytes
ucp1	0.2505341	6.44E-24	2	Hepatocytes
hpx	0.44955698	2.15E-29	3	Hepatocytes
fabp10a	0.30927872	5.54E-50	4	Hepatocytes
ucp1	0.32230532	1.08E-24	4	Hepatocytes
adh8a	0.29238025	6.70E-07	7	Hepatocytes
fbp1b	0.26385816	8.03E-08	7	Hepatocytes
gstt1a	0.25554857	1.45E-07	7	Hepatocytes
si:ch211-270n8.1	0.37144675	3.89E-05	7	Hepatocytes
zgc:123103	0.32403081	5.88E-09	7	Hepatocytes
coro1a	1.56441663	4.36E-230	5	Immune cells
coro1a	2.03229575	2.77E-95	8	Immune cells
ctss2.1	2.51451992	0	5	Immune cells
ctss2.2	2.53690115	7.47E-110	5	Immune cells
mfap4	1.83148943	3.63E-289	5	Immune cells
MFAP4 (1 of many).2	2.52132639	0	5	Immune cells
mpeg1.1	2.21584506	0	5	Immune cells
wasb	1.43659107	1.07E-188	5	Immune cells
wasb	2.09590626	3.48E-99	8	Immune cells
actb2	0.36766069	3.77E-11	6	Hepatic stellate/ endothelial cells
krt8	0.62566416	1.73E-11	6	Hepatic stellate/ endothelial cells

Table S22: Markers used to predict cluster composition in t-SNE plot consisting of livers from HCC (HepABC), HCC (CreLox) and No HCC zebrafish.

Click here to Download Table S22

Biology Open • Supplementary information
